# Establishing the clinical utility of integrated quasistatic acoustic tweezing thromboelastometry for blood coagulation analysis

**DOI:** 10.1016/j.jtha.2025.12.020

**Published:** 2026-01-16

**Authors:** Huy Q. Pham, Collette Barnor, Trishita Paul, Elizabeth M. Cummins, Daniel Arango, Shaun Yockelson, Damir B. Khismatullin

**Affiliations:** 1 Department of Biomedical Engineering, Tulane University, New Orleans, Louisiana, USA; 2 Department of Anesthesiology and Perioperative Medicine, Ochsner Medical Center, New Orleans, Louisiana, USA; 3 Levisonics Inc, New Orleans, Louisiana, USA

**Keywords:** blood coagulation tests, blood coagulation disorders, fibrinogen, heparin, liver transplantation

## Abstract

**Background:**

Rapid and accurate coagulation analysis is essential for managing critical care and surgical patients, patients with coagulation disorders, and patients with cardiovascular disease on anticoagulant therapy. Traditional assays often require large sample volumes, posing iatrogenic risks in vulnerable populations, including pediatric and elderly patients. Integrated quasistatic acoustic tweezing thromboelastometry (i-QATT) offers a comprehensive, noncontact assessment of coagulation using a 4- to 6-μL drop of blood that minimizes diagnostic errors associated with sample-container surface interactions.

**Objectives:**

This study aims to assess the preliminary clinical utility of the i-QATT technique.

**Methods:**

Using blood samples from healthy volunteers and liver transplant patients, as well as control plasmas, we established the reference ranges for i-QATT coagulation parameters and validated them across several abnormal conditions, including single-factor deficiencies, abnormal fibrinogen levels, unfractionated heparin anticoagulation, and coagulopathy.

**Results:**

i-QATT identified coagulation abnormalities within 3 to 8 minutes. Its parameters showed strong correlations with gold-standard assay data, for example, activated partial thromboplastin time (*r* ≥0.81), international normalized ratio (*r* ≥ 0.74), thromboelastography (TEG) citrated kaolin channel parameters (R, K, α, and maximum width of TEG tracing [MA]; *r* ≥ 0.65), citrated rapid TEG (MA; *r* = 0.64), and TEG citrated functional fibrinogen (MA; *r* ≥ 0.64). i-QATT data demonstrated a near-linear response to unfractionated heparin concentrations spanning the prophylactic and therapeutic ranges (0–1 IU/mL; *R*^2^ = 0.98), and the anticoagulation reversal by heparinase was reliably detected. i-QATT accurately assessed the functional level of fibrinogen and platelet activity in normal and abnormal blood samples.

**Conclusions:**

This study positions i-QATT as a highly sensitive, rapid, and ultra–low-volume alternative to conventional coagulation assays, with strong potential to transform point-of-care diagnostics across a wide range of clinical settings.

## 1 | INTRODUCTION

Blood coagulation analysis is the first step in assessment of bleeding/thrombosis risks in critical care patients [1,2], patients with inherited or acquired coagulation disorders [1,3,4], and patients on anticoagulant therapy or hemodialysis [5,6]. It is also used to guide intraoperative blood transfusion [7]. Most coagulation tests performed in hospitals are blood plasma turbidimetric tests such as prothrombin time (PT)/international normalized ratio (INR) and activated partial thromboplastin time (APTT), which measure the onset of fibrin formation. While useful for screening single-factor deficiencies in the intrinsic and extrinsic coagulation pathways, these tests have limited sensitivity to abnormalities in the common pathway or in later stages such as fibrinolysis [8]. In perioperative settings, their capability to measure coagulopathy- and transfusion-induced hemostatic changes remains questionable [9].

Whole blood (WB) viscoelastic hemostatic assays (VHAs), namely thromboelastography (TEG) and rotational thromboelastometry (ROTEM), are existing alternatives to PT/APTT. They measure the changes in clot firmness during fibrin crosslinking and fibrinolysis. TEG/ROTEM are useful for detecting hypocoagulable and hypercoagulable states and can guide blood transfusion [10,11]. Improved patient outcomes have been reported with TEG/ROTEM-guided bleeding management algorithms, with a 1A recommendation received in recent clinical guidelines [12–14]. While TEG has limited reliability in detecting warfarin (a vitamin K antagonist) due to the absence of a tissue factor–activated assay [15,16], ROTEM EXTEM clotting time (CT) correlates very well (*r* = 0.86) with INR in patients treated with vitamin K antagonists [17,18]. However, these techniques are not standardized for coagulation measurements [19–21].

Current VHAs operate with relatively large blood sample volumes. TEG 5000 and ROTEM delta require 0.36 and 0.30 mL of blood per test, respectively, with multiple tests typically ordered [22,23]. TEG 6s needs 340 μL for its 4-channel cartridge [24]. Blood collection for these tests may cause iatrogenic anemia in neonates and small children [25], critical care patients [26], and the elderly [27]. The existing coagulation analyzers also expose blood samples to artificial surfaces, which may influence coagulation kinetics and contribute to variability [28]. Drop-of-blood acoustic tweezing coagulometry addresses these issues. One of its techniques, referred to as integrated quasistatic acoustic tweezing thromboelastometry (i-QATT), can perform containerless turbidimetric and elastometric measurements of blood coagulation using a drop of WB/blood plasma with a volume of 4 to 6 μL, nearly 100 times less than the volume required by abovementioned techniques. i-QATT analysis provides information about the kinetics of fibrin formation, coagulation onset, coagulation propagation (liquid to solid change) and saturation (clot fully formed), and clot degradation (fibrinolysis) [29,30]. i-QATT provides a global coagulation profile using a minimal sample volume, but its clinical utility remains uncertain.

In this article, we establish the reference ranges of i-QATT coagulation parameters and test them under various abnormal clinical conditions, including single-factor deficiencies, abnormal fibrinogen levels, heparin anticoagulation, and liver transplantation and associated coagulopathy. Furthermore, we perform correlation analysis between i-QATT and gold standard coagulation assays (APTT, INR, and TEG) using WB and plasma samples from healthy volunteers and liver transplant patients, as well as commercial plasma samples.

## 2 | METHODS

### 2.1 | Reagents and commercial blood samples

PT (Thromboplastin-D) and APTT (APTT-XL) reagents were obtained from Fisher Scientific. Calcium chloride, heparin sodium salt (unfractionated heparin [UFH]), fibrinogen, cytochalasin-D (CytD), all in powder form, were purchased from Sigma-Aldrich. Heparinase I (Dade Hepzyme) was obtained from Siemens Healthcare Diagnostics.

Platelet-poor plasma (PPP) samples deficient in factor (F)VII and FIX and half-depleted in all factors (coagulopathy simulation) were purchased from Affinity Biologicals. PPP deficient in FX and SysCOR 40 Control Kit, containing borderline factor assay control (B-FACT) and positive lupus anticoagulant PPP, were obtained from George King Bio-Medical.

### 2.2 | Collection and preparation of blood samples from healthy volunteers and liver transplant patients

The protocols for blood collection from healthy and liver transplant subjects were approved by the Institutional Review Boards of Tulane University (No. 520566 and 944474) and Ochsner Medical Center (No. 2020.245). In total, 34 healthy volunteers (16 males and 18 females), aged 19 to 42 years, and 8 liver transplant recipients (6 males and 2 females), aged 29 to 67 years, were enrolled in this study. The racial composition of enrolled subjects was as follows: 43% White, 26% African, 24% Asian, 5% Hispanic, and 2% multiracial. Subjects with a known history of liver disease or coagulopathy, those who smoke, those taking medications that affect the coagulation system, and individuals with a BMI of 35 kg/m^2^ or higher were excluded from the healthy group. Each healthy volunteer donated 22.8 mL of blood via venipuncture collected into 4 citrated (2.7 mL each) and 2 K_2_EDTA tubes (6 mL each; BD). These tubes were selected because they are standard for coagulation analysis in adult health care. Blood for i-QATT measurements can potentially be collected in microliter-volume tubes, which are commercially unavailable at present. Half of the collected sample were sent to Tulane Coagulation Laboratory for PT/INR, APTT, fibrinogen, and platelet count tests. The remaining sample was used for i-QATT measurements within 4 hours of collection.

Citrated WB samples (5.4 mL in two 2.7-mL tubes) were obtained from liver transplant patients: (1) immediately after induction of anesthesia (baseline time point), (2) at 5 minutes after arterial reperfusion (artery time point), and (3) during the biliary reconstruction (final time point). The samples were collected from an indwelling radial artery catheter via a closed-circuit sampling system. One of the tubes was used for standard-of-care TEG testing at each time point, and the WB sample in the other tube was tested by i-QATT within 4 hours of collection. After measuring the latter sample by i-QATT, the rest was centrifuged at 3500 × *g* for 10 minutes or twice at 1500 × *g* for 15 minutes to isolate PPP, which was stored in a 1.5-mL microcentrifuge tube or cryovial at −80 °C until tested.

### 2.3 | Coagulation tests

#### 2.3.1 | i-QATT analysis

The i-QATT analysis was conducted using an acoustic tweezing system (Levisonics). The intrinsic pathway activator was prepared by diluting 3.7 mg CaCl_2_ into 500 μL of the APTT reagent solution. For the extrinsic pathway activation, CaCl_2_ was diluted in phosphate-buffered saline (PBS) to 0.2 M concentration. The PT reagent powder was reconstituted in 4 mL PBS per manufacturer instructions. A mixture of 36 μL of the CaCl_2_ solution and 4 μL of the PT reagent solution was then prepared.

Prior to i-QATT test, an 18-μL aliquot of WB or PPP was withdrawn from the blood collection tube or microcentrifuge tube, respectively, and transferred to a 0.2-mL polymerase chain reaction tube, where it was incubated at 37 °C for 5 minutes and then mixed with 6 μL of the intrinsic pathway activator or 2 μL of the extrinsic pathway activator. A 6-μL drop of the resulting solution was deployed into the acoustic tweezing device using a 0.2- to 10-μL single-channel electronic pipette (CAPP Maestro M10–1) with a gel-loading tip.

**FIGURE 1:**
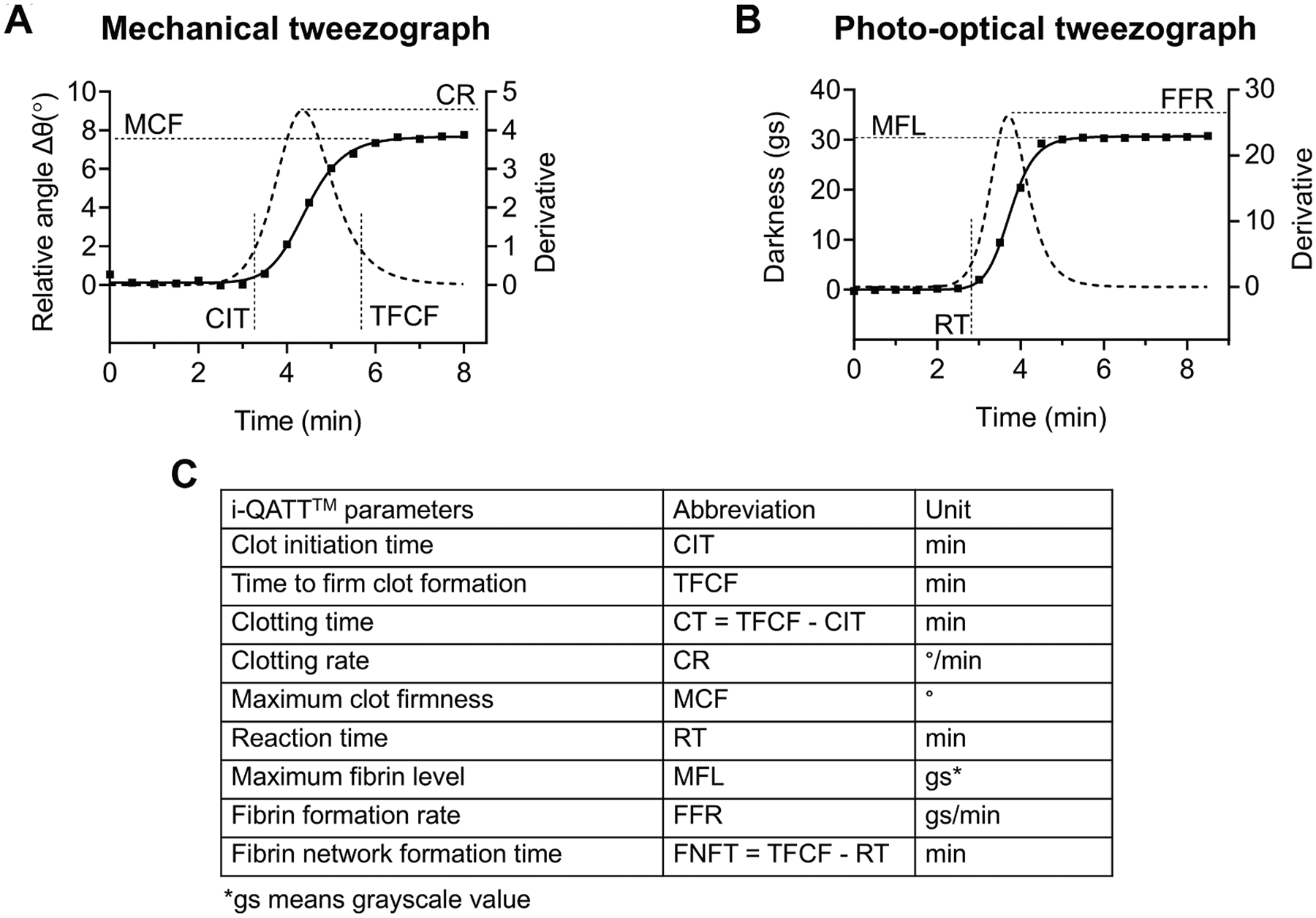
i-QATT graphical outputs (A, B) and parameters (C). i-QATT, integrated quasistatic acoustic tweezing thromboelastometry.

The blood drop was first maintained at an aspect ratio of 1.2 (resting state) and then subjected to quasistatic deformation via a 30-second pressure sweep [29,30]. The vertical location (extensional stress), aspect ratio (strain), and central light intensity of the drop were assessed from the drop images collected every 0.3 seconds. We measured the slope of the linear part of location vs aspect ratio curve for each pressure sweep to assess the blood firmness (elasticity) and plotted the slope as a function of time (sweep number × 30 seconds). The resulting graph, referred to as mechanical tweezograph, is analogous to outputs from TEG, ROTEM, and other VHA techniques. For blood plasma analysis, the light intensity in grayscale values (0–255), representing blood turbidity, was also plotted as a function of time and referred to as photo-optical tweezograph. Blood coagulation parameters were obtained from analysis of mechanical or photo-optical tweezographs (Figure 1) using a custom MATLAB code (MathWorks). i-QATT showed consistent performance across broad ranges of humidity (60%-99%) and temperature (room to body temperature), and vibration levels exceeding those typical of hospital environments.

To assess fibrinogen and platelet contributions to clot firmness, WB samples were incubated with CytD at 9:1 (v/v) ratio (4 μM final concentration) at room temperature for 10 minutes following the previously established protocol [29]. The same effect was observed with a 1-minute incubation (Supplementary Figure S1A), with no impact of the CytD buffer (Supplementary Figure S1B).

UFH was diluted in PBS to 100 IU/mL stock solution. Healthy volunteer PPP and B-FACT PPP were mixed with UFH-in-PBS solution to achieve final UFH concentrations ranging from 0.1 to 0.5 IU/mL. WB samples were spiked with UFH at 0.5 IU/mL concentration. A 20:1 (v/v) sample-to-UFH ratio was maintained for both PPP and WB. For UFH reversal, heparinized PPP and WB samples were treated with heparinase at a 10:1 (v/v) ratio (0.11 IU/μL final concentration). A fibrinogen stock (6669 mg/dL) dissolved in PBS was added to PPP or WB samples to increase fibrinogen levels by 271 mg/dL (PPP and WB) or 667 mg/dL (WB).

#### 2.3.2 | Standard plasma coagulation tests (INR and APTT) and TEG

INR and APTT values were provided by manufacturers or measured by Tulane Coagulation Laboratory (healthy volunteer samples). INR was also measured at each blood collection time point for liver transplant patients. Liver transplant samples were analyzed using 4 TEG 6s assays (Haemonetics): citrated kaolin (CK), citrated rapid TEG (CRT), citrated kaolin with heparinase (CKH), and citrated functional fibrinogen (CFF) [31]. The following TEG parameters were compared with i-QATT parameters: R (reaction time), K (kinetic time), α (rate of clot formation), and maximum width of TEG tracing (MA) [32].

### 2.4 | Data analysis

Statistical analysis was conducted using Prism software (GraphPad). Data were presented as mean ± standard error of the mean (SEM). The *P* values were calculated using the 2-tailed unpaired *t*-test for normally distributed data, the Mann–Whitney U-test for nonnormally distributed data, and 1-way analysis of variance (ANOVA) for comparing more than 2 groups. Data normality was assessed with the Shapiro–Wilk test. Statistical significance was set at *P* < .05. The goodness of the fit for dose-response data was assessed by the coefficient of determination *R*^2^.

The correlation between the i-QATT and gold standard assay (INR, APTT, and TEG) data was evaluated using the Pearson correlation coefficient (*r*_*p*_) for normally distributed data or the Spearman rank correlation coefficient (*r*_*s*_) for nonnormally distributed data. The correlation strength (*r*_*p*_ or *r*_*s*_) was defined as follows: <0.4, very weak/weak; 0.4 to 0.59, moderate; 0.60 to 0.79, strong; and 0.80 to 1.0, very strong [33].

Reference ranges of i-QATT coagulation parameters were established using Reference Value Advisor software [34]. For normally distributed data assessed with the Anderson-Darling test, the reference range was determined as follows:

(1) $$\left[\text{lower limit};\text{upper limit}\right]=\left[\hat{m}-t_{N-1}^{0.975}\hat{\sigma }\sqrt{\frac{N+1}{N}};\hat{m}+t_{N-1}^{0.975}\hat{\sigma }\sqrt{\frac{N+1}{N}}\right]$$

where $\hat{m}$ is the sample mean; *N*, the sample size; $t_{N-1}^{0.975}$, the 0.975 quantile of a Student distribution with *N* − 1 degrees of freedom; and $\hat{\sigma }$, the sample standard deviation (SD). Nonnormally distributed data were transformed using the generalized Box-Cox method before calculating the reference range with Eq. (1). Precision of the lower and upper limits was calculated using 90% confidence intervals (CIs).

Further details on materials and methods are provided in the Supplementary Methods section of the Supplementary Information.

## 3 | RESULTS

### 3.1 | i-QATT can quickly detect coagulation abnormalities

**FIGURE 2:**
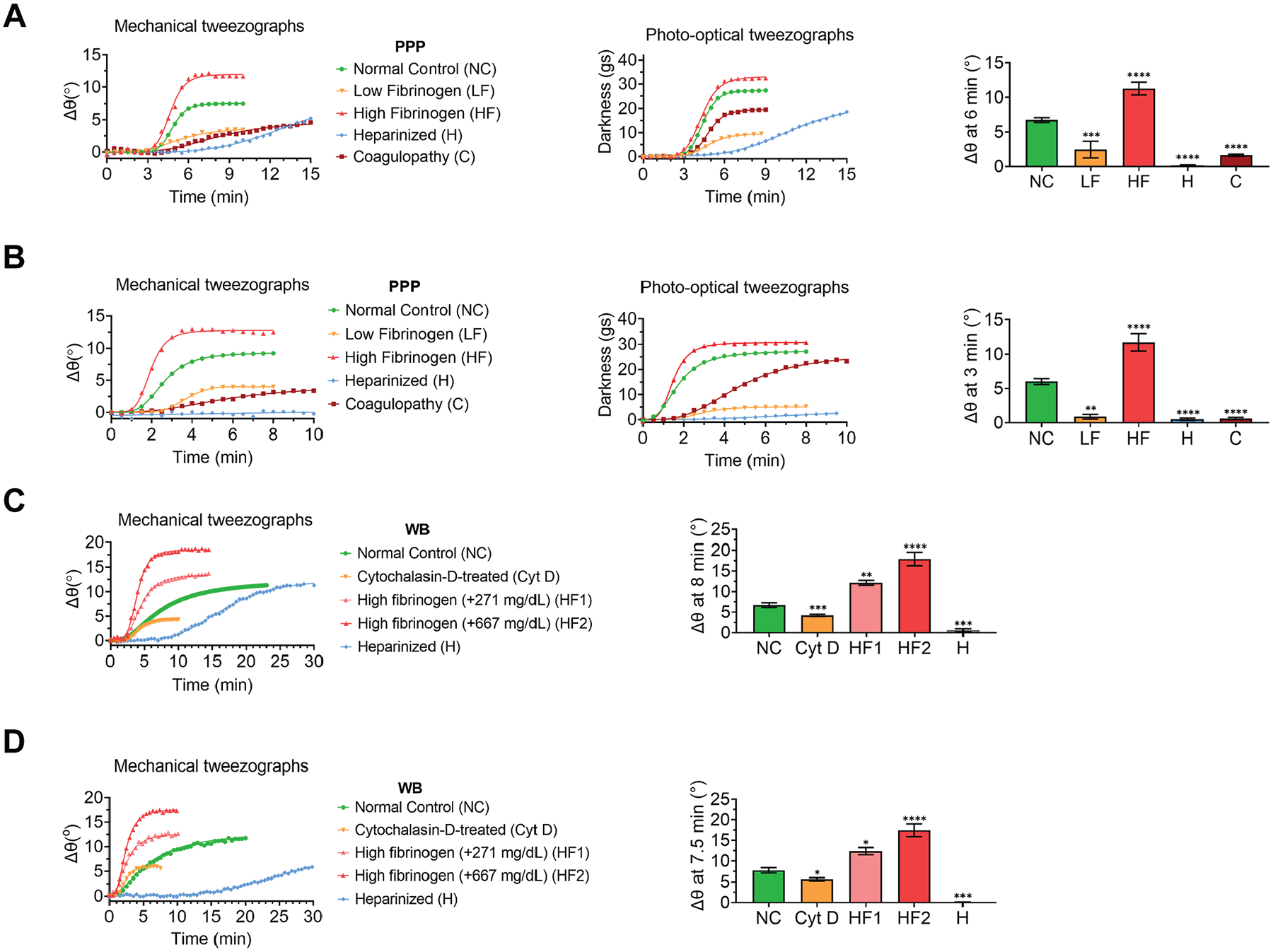
Representative i-QATT tweezographs (mechanical and photo-optical) and clot firmness values under various hemostatic conditions. (A) Intrinsic-activated PPP at 6 minutes: normal (*n* = 34), low fibrinogen (120 ± 15 mg/dL, *n* = 3), high fibrinogen (546 ± 13 mg/dL, *n* = 5), heparin-treated (0.5 IU/mL, *n* = 5), and coagulopathic (*n* = 4) samples. (B) Extrinsic-activated PPP at 3 minutes: normal (*n* = 34), low fibrinogen (101 ± 13 mg/dL, *n* = 4), high fibrinogen (553 ± 14 mg/dL, *n* = 4), heparin-treated (0.5 IU/mL, *n* = 5), and coagulopathy (*n* = 3) samples. (C) Intrinsic-activated WB at 8 minutes: normal (*n* = 33), cytochalasin-D–treated (4 μM, *n* = 33), high fibrinogen (558 ± 23 mg/dL, *n* = 3; 983 ± 24 mg/dL, *n* = 6), and heparin-treated (0.5 IU/mL, *n* = 4) samples. (D) Extrinsic-activated WB at 7.5 minutes: normal (*n* = 30), cytochalasin-D–treated (4 μM, *n* = 19), high fibrinogen (519 ± 20 mg/dL, *n* = 4; 993 ± 26 mg/dL, *n* = 5), and heparin-treated (0.5 IU/mL, *n* = 3) samples. Values are mean ± SEM; *P < .05; **P < .01; ***P < .001; ****P < .0001. i-QATT, integrated quasistatic acoustic tweezing thromboelastometry; PPP, platelet-poor plasma; WB, whole blood.

Timely assessment of coagulation status is critical for guiding clinical interventions in trauma and critical care and for monitoring anticoagulant therapy. Kaolin-activated TEG is constrained by a delayed coagulation onset, taking up to 9 minutes in normal WB as measured by R-time [35], thus requiring more than 10 minutes to differentiate abnormal from normal clotting. Rapid-TEG and ROTEM assays such as EXTEM and FIBTEM provide faster readouts [15,36]. In the i-QATT technique, changes in graphical outputs became detectable in within 6 minutes in normal PPP or WB (Figure 2), including a 1-minute incubation with coagulation activators and other reagents. Significant differences in clot firmness between normal and abnormal samples were observed as early as 6 minutes in intrinsic pathway–activated PPP, 3 minutes in extrinsic pathway–activated PPP, and 8 minutes in WB activated via either pathway (Figure 2, right). The time required to detect significant differences in WB with i-QATT is comparable with that with ROTEM. The i-QATT mechanical tweezographs of intrinsic pathway–activated PPP exhibited prolonged coagulation for heparinized and coagulopathic samples and hypercoagulability and hypocoagulability for high- and low-fibrinogen samples, respectively (Figure 2A, left). Similar effects were seen in mechanical tweezographs of extrinsic pathway–activated PPP (Figure 2B, left). Photo-optical data demonstrated sensitivity to fibrinogen and heparin; however, prolonged coagulation in coagulopathic samples was only seen for the extrinsic pathway of coagulation (Figure 2A, B, middle). Coagulopathic samples exhibited normal coagulation in the intrinsic pathway because of the compensatory effect of FVIII and FIX, which were only half-depleted in those samples. The WB mechanical tweezographs showed delayed coagulation for heparinized samples, but clotting was accelerated for normal WB spiked with fibrinogen (Figure 2C, D). Clot firmness was sensitive to fibrinogen concentration and decreased with CytD treatment. Heparin had a more pronounced effect on extrinsic pathway data than that on the intrinsic one (compare Figure 2C, D).

### 3.2 | i-QATT is highly sensitive to factor deficiency, heparin anticoagulation, and fibrinogen concentration

Blood coagulation involves numerous components, including platelets, coagulation factors, and inhibitors, whose concentrations and activities naturally vary among healthy individuals due to genetic, dietary, environmental, and physiological factors. These biological differences led to the variability in i-QATT graphical outputs (Supplementary Figure S2). Reference ranges of i-QATT parameters, extracted from these graphs, are listed in Table for PPP and WB samples. The parameters representing coagulation onset exhibited narrower ranges than those for propagation and saturation. In particular, PPP clotting initiation time (CIT) and reaction time (RT) exhibited range widths (difference between upper and lower limits) of 2.01 and 1.81 minutes for the intrinsic pathway and 1.33 and 0.74 minutes for the extrinsic pathway, respectively. Time to firm clot formation (TFCF) showed broader ranges than CIT and RT (3.13 minutes for intrinsic and 3.65 minutes for extrinsic) yet remained narrower than clotting rate (CR), maximum clot firmness (MCF), fibrin formation rate (FFR), and maximum fibrin level (MFL). The widest reference ranges were observed for PPP MFL and FFR, with widths of 22.54 grayscale (gs) and 25.41 gs/min for the intrinsic pathway and 23.58 gs and 17.60 gs/min for the extrinsic pathway, respectively. Mechanical parameters MCF and CR of PPP samples showed intermediate variability, with range widths equal to 6.62° and 8.67°/min for the intrinsic pathway and 7.20° and 6.70°/min for the extrinsic pathway, respectively.

The presence of platelets and other cellular components in WB did not significantly impact CIT (range width of 3.03 minutes for the intrinsic pathway and 1.84 minutes for the extrinsic pathway) but introduced greater variability in late-stage parameters TFCF and MCF (Table). TFCF and MCF range widths were 12.12 minutes and 15.04° for the intrinsic pathway and 15.52 minutes and 16.27° for the extrinsic pathway, respectively. CR showed relatively low variability (2.44°/min for intrinsic and 3.16°/min for extrinsic). The marked reduction in MCF variability in CytD-treated WB (õne-third of untreated WB: 5.64 vs 15.04 for intrinsic and 6.64 vs 16.27 for extrinsic) underscores platelet function as the dominant source of intersubject variability.

**FIGURE 3:**
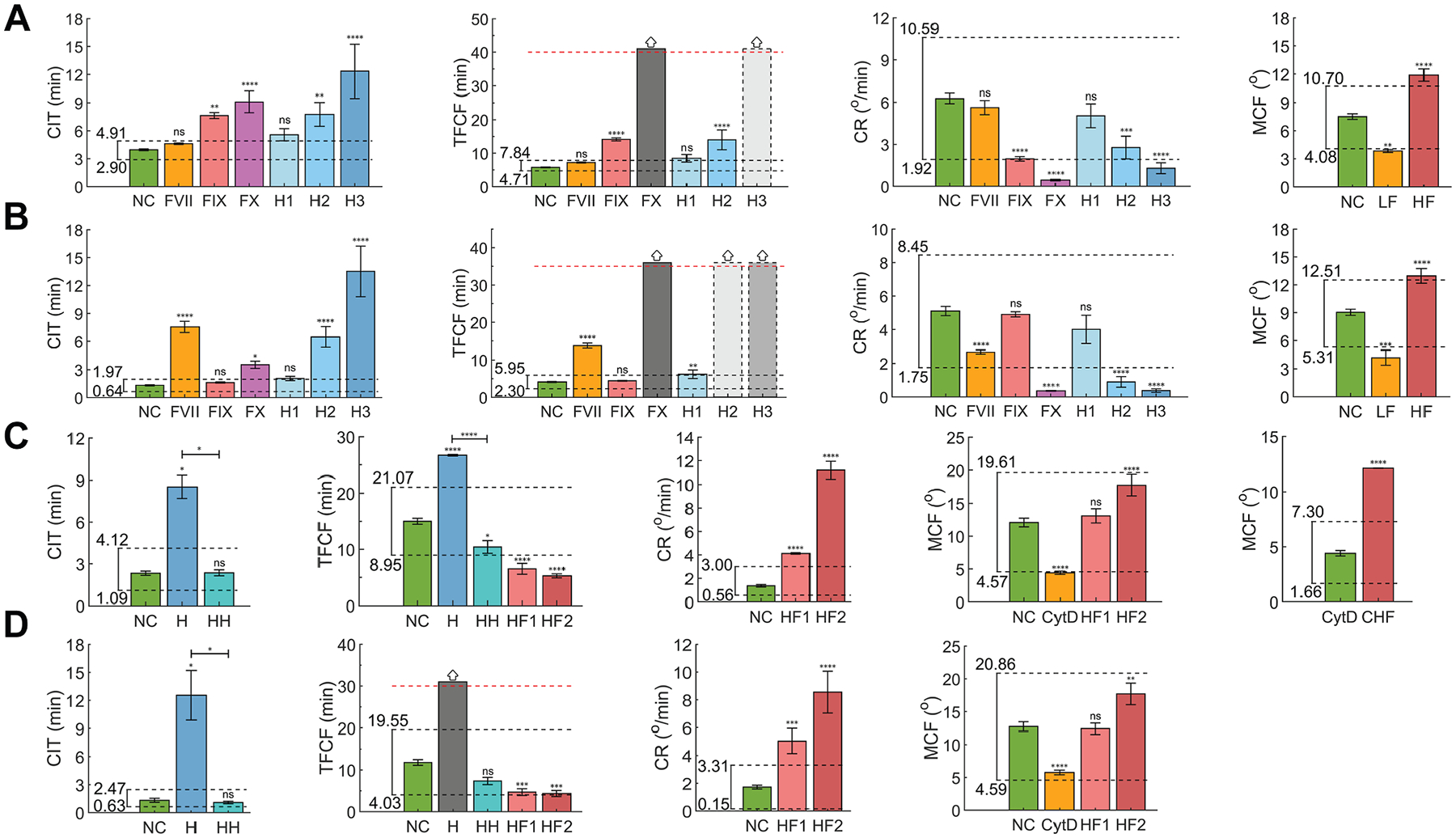
Validation of i-QATT reference ranges using PPP and WB samples under various coagulation conditions. (A, B) PPP samples under normal control (NC); factor (F)VII-, FIX-, and FX-deficient; heparin-spiked (H1, 0.1 IU/mL; H2, 0.3 IU/mL; H3, 0.5 IU/mL); and low fibrinogen (LF; 120 ± 15 mg/dL for intrinsic; 101 ± 13 mg/dL for extrinsic) or high fibrinogen (HF; final concentration of 546 ± 13 mg/dL for intrinsic; 553 ± 14 mg/dL for extrinsic) conditions, with activation via the intrinsic (A) or extrinsic (B) pathway. (C, D) WB samples under NC, heparin-treated (H, 0.5 IU/mL), heparinase-treated following heparinization (HH), cytochalasin-D–treated (CytD; 4 μM), and HF (HF1, 558 ± 23 and HF2, 983 ± 24 mg/dL for intrinsic; HF1, 519 ± 20 and HF2, 993 ± 26 mg/dL for extrinsic) conditions, with activation via the intrinsic (C) or extrinsic (D) pathway. CHF is cytochalasin-D–treated WB spiked with fibrinogen at a final concentration of 939 ± 15 mg/dL. Values are mean ± SEM (*n* = 3–34); *P < .05; **P < .01; ***P < .001; ****P < .0001. i-QATT, integrated quasistatic acoustic tweezing thromboelastometry; PPP, platelet-poor plasma; WB, whole blood.

To validate the reference ranges, we performed i-QATT measurements on factor-deficient, heparinized, and abnormal fibrinogen PPP samples as well as on abnormal WB samples including heparinized, heparinized followed by heparinase treatment, CytD-treated, fibrinogen-spiked, and CytD-treated samples with subsequent fibrinogen spiking. Coagulation data for these samples are shown in Figure 3.

As anticipated, for the intrinsic pathway activation (Figure 3A), FIX- and FX-deficient PPP, but not FVII-deficient PPP, exhibited elevated CIT values exceeding the reference range upper limit, with statistically significant differences from normal PPP. UFH-spiked samples (≥0.1 IU/mL) exhibited abnormally high CIT, with statistically significant differences at ≥0.3 IU/mL. TFCF showed similar trends; however, coagulation in FX-deficient PPP and in PPP exposed to high concentrations of UFH was often markedly delayed, yielding unmeasurably high TFCF values (gray bars in the second plot in Figure 3A). Abnormally low CR was also detected in FIX- and FX-deficient PPP and UFH-spiked PPP (0.5 IU/mL). MCF fell below the lower reference limit in low-fibrinogen PPP and exceeded the upper limit in high-fibrinogen PPP, with both conditions showing statistically significant differences compared with those of normal control PPP. RT and FFR also accurately predicted normal coagulation in FVII-deficient PPP and delayed coagulation in FIX- and FX-deficient PPP and heparinized PPP (Supplementary Figure S3A).

Factor IX deficiency led to normal values of coagulation parameters in extrinsic pathway–activated PPP, while FVII deficiency led to CIT, TFCF, RT, and FFR values outside their reference ranges (Figure 3B; Supplementary Figure S3B). CR in FVII-deficient PPP was significantly lower than that in normal but remained within the range. The UFH effect was much stronger in the extrinsic pathway than that in the intrinsic one, yielding unmeasurably high TFCF values at relatively low concentrations of UFH. CIT, CR, and FFR also showed more pronounced, nonlinear changes with UFH concentration. This indicates that i-QATT better predicts heparin dose effects in intrinsic pathway–activated samples, where the UFH concentration dependence is more linear (compare Figure 3A, B; Supplementary Figure S3A, B). The fibrinogen effect on MCF was comparable between the extrinsic and intrinsic pathway–activated PPP samples (last plot in Figure 3B).

UFH at 0.5 IU/mL strongly affected intrinsic coagulation in WB samples, with CIT values twice the upper reference limit. This prolonged coagulation was neutralized by heparinase (first plot in Figure 3C). The anticoagulation effect of UFH and its neutralization by heparinase were also measurable using WB TFCF (second plot in Figure 3C). TFCF and CR were abnormally low and high, respectively, in fibrinogen-spiked WB samples (second and third plots in Figure 3C). While MCF was sensitive to fibrinogen concentration in PPP, intrinsic pathway–activated WB samples spiked with fibrinogen at an above-normal final concentration maintained MCF values within the reference range. When the platelet activity was blocked by CytD, MCF dropped below the lower reference limit in normal WB samples (fourth plot in Figure 3C) and elevated above the upper limit in fibrinogen-spiked WB samples (fifth plot in Figure 3C).

In extrinsic pathway–activated WB samples, CIT increased 5-fold from the upper reference limit upon exposure to 0.5 IU/mL UFH (first plot in Figure 3D). This strong effect was also observed for TFCF (second plot in Figure 3D). As in the case of the intrinsic pathway, both CIT and TFCF showed the reversal effect of heparinase on UFH anticoagulation. Extrinsic TFCF was reduced in fibrinogen-spiked WB samples but remained within the reference range, while CR increased above the range (third plot in Figure 3D).

### 3.3 | i-QATT strongly correlates with standard plasma tests and WB TEG

**FIGURE 4:**
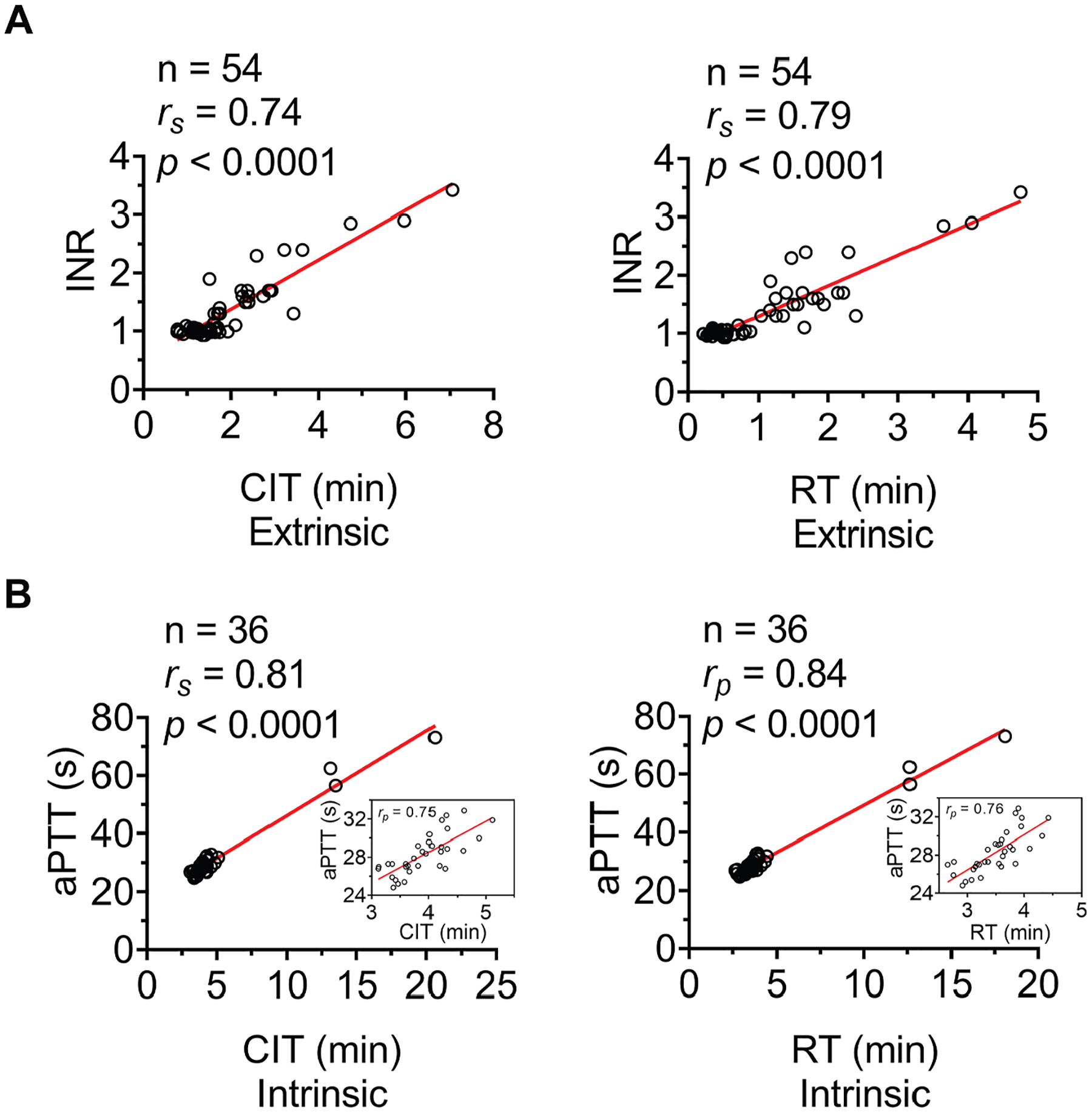
Correlation between i-QATT and standard plasma coagulation tests (INR and APTT) for normal, liver transplant, and abnormal control PPP samples. (A) Extrinsic CIT and RT vs INR. Samples analyzed include 31 healthy, 21 liver transplant, and 2 abnormal control PPP samples. (B) Intrinsic CIT and RT vs APTT. Insets show the correlation data for healthy subjects. Samples analyzed include 33 healthy and 3 for abnormal control PPP samples. Note that APTT measurements were not collected for liver transplant patients. Insets show the correlation data for healthy subjects. APTT, activated partial thromboplastin time; CIT, clot initiation time; INR, international normalized ratio; i-QATT, integrated quasistatic acoustic tweezing thromboelastometry; PPP, platelet-poor plasma; RT, reaction time.

To evaluate i-QATT against gold standard tests, we conducted correlation analyses between i-QATT parameters and INR, APTT, and TEG parameters from various assay channels. For normal, abnormal, and liver transplant PPP samples activated via the extrinsic pathway, i-QATT CIT and RT strongly correlated with INR (*r* ≥ 0.74) (Figure 4A). A very strong correlation was observed between APTT values and intrinsic i-QATT RT or CIT in normal and abnormal PPP samples (*r* ≥ 0.81) (Figure 4B). TFCF also had a very strong correlation with APTT (*r* = 0.80), while a significant but weak correlation (*r* < 0.40) was seen for CT and fibrin network formation time (FNFT) vs APTT (Supplementary Figure S4A).

**FIGURE 5:**
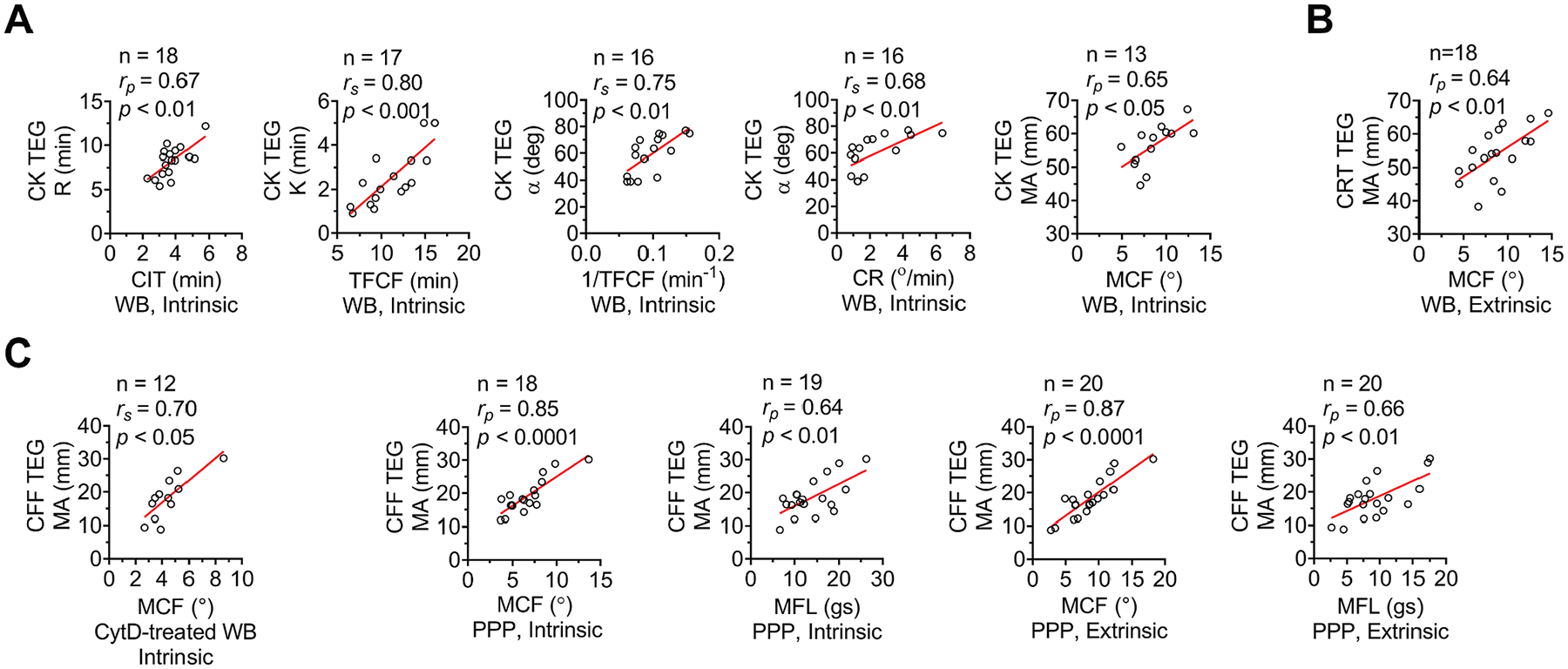
Correlation between i-QATT parameters and TEG parameters for citrated kaolin (CK), citrated rapid TEG (CRT), and citrated functional fibrinogen (CFF) channels. (A) Intrinsic i-QATT vs CK TEG: CIT vs R, TFCF vs K, 1/TFCF and CR vs α, and MCF vs MA. (B) Extrinsic i-QATT vs CRT TEG: MCF vs MA. (C) Intrinsic and extrinsic i-QATT vs CFF TEG. Intrinsic CytD-treated WB MCF vs MA. Intrinsic PPP MCF and MFL vs MA. Extrinsic PPP MCF and MFL vs MA. CIT, clot initiation time; CR, clotting rate; CytD, cytochalasin-D; MA, maximum width of TEG tracing; MCF, maximum clot firmness; MFL, maximum fibrin level; i-QATT, integrated quasistatic acoustic tweezing thromboelastometry; PPP, platelet-poor plasma; TEG, thromboelastography; TFCF, time to firm clot formation; WB, whole blood.

The i-QATT parameters CIT, TFCF, CR, and MCF measured in liver transplant WB samples activated via the intrinsic pathway also showed a strong correlation with R, K, α, and MA of the TEG CK channel (*r* ≥ 0.65) (Figure 5A). A close association was seen between i-QATT MCF of extrinsic pathway–activated WB and MA of TEG CRT channel (*r* = 0.64) (Figure 5B).

Intrinsic i-QATT MCF of liver transplant WB treated with CytD was strongly correlated with MA of the TEG CFF channel (*r* = 0.7) (first plot in Figure 5C). Note that the platelet activity was blocked in the CFF channel, similar to the effect of CytD. For PPP samples, i-QATT correlation with TEG CFF was even stronger: *r* = 0.85 for intrinsic MCF vs MA and 0.87 for extrinsic MCF vs MA (second and fourth plots in Figure 5C). Strong correlation was also established between i-QATT MFL and MA in the TEG CFF channel: *r* =0.64 and 0.66 for intrinsic and extrinsic pathway–activated PPP samples, respectively (third and fifth plots in Figure 5C).

### 3.4 | i-QATT is sensitive to fibrinogen concentration and platelet activity

**FIGURE 6:**
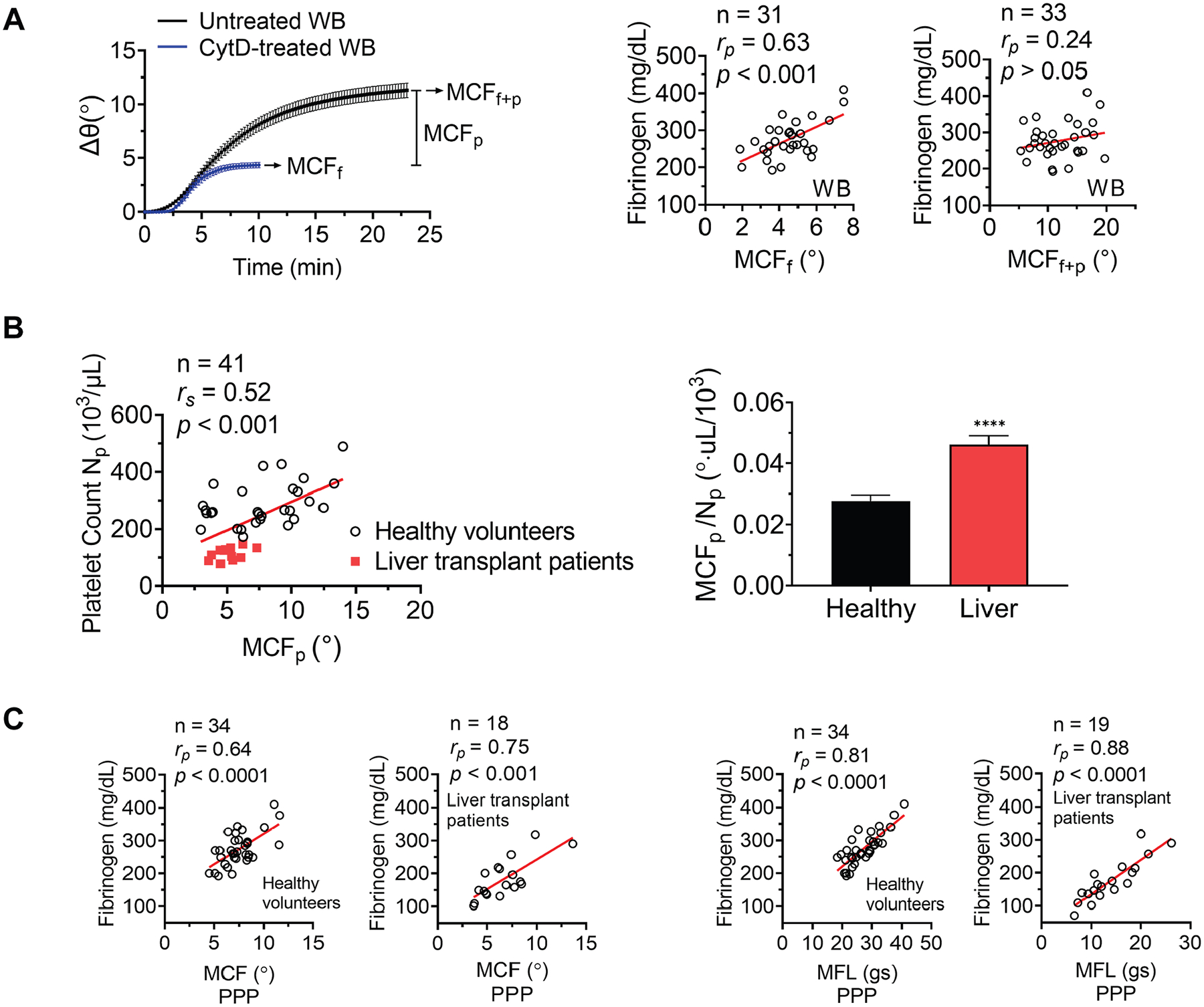
i-QATT functional fibrinogen assay using WB and PPP samples treated via the intrinsic pathway. (A) Mechanical tweezographs of untreated and CytD-treated WB samples (left). The difference between untreated WB MCF (MCF_f+p_) and CytD-treated WB MCF (MCF_f_) allows to measure the contribution of platelets to clot firmness (MCF_p_). Shown also are correlation between MCF and fibrinogen concentration in CytD-treated WB (middle) and untreated WB (right). (B) Correlation between MCF_p_ and platelet count N_p_ for healthy and liver transplant samples (left) and the ratio of MCF_p_ to N_p_ across both groups (right), demonstrating platelet hyperactivity in liver transplant patients. (C) Correlation plots of MCF (left) and MFL (right) vs fibrinogen concentration in PPP samples from healthy volunteers and liver transplant patients. CytD, cytochalasin-D; MCF, maximum clot firmness; MFL, maximum fibrin level; i-QATT, integrated quasistatic acoustic tweezing thromboelastometry; PPP, platelet-poor plasma; WB, whole blood.

Both fibrinogen and platelets contribute to WB clot firmness. By inhibiting platelet function with CytD, the contribution of fibrinogen to clot firmness can be assessed. When activated via the intrinsic pathway, normal WB samples spiked with CytD were much softer than untreated WB samples, leading to lower MCF values (Figure 6A, left). This low MCF, referred to as MCF_f_, had a strong correlation (*r* = 0.63) with Clauss fibrinogen (middle plot), while a very weak correlation (*r* = 0.24) was observed for untreated WB MCF (MCF_f+p_, right plot).

The difference between MCF_f+p_ and MCF_f_ quantifies the platelet contribution to clot firmness, referred to as MCF_p_ (Figure 6A, left). Using this measure, it is possible to assess platelet activity in different human subjects. As seen in Figure 6B (left), liver transplant patient data lie below the platelet count vs MCF_p_ regression line (red), indicating increased platelet activity. Similarly, the ratio of MCF_p_ to platelet count predicted a significantly higher activity of platelets in liver transplant patients than that in healthy volunteers (Figure 6B, right).

i-QATT provides direct measurement of the functional level of fibrinogen in PPP through MCF and MFL (Figure 6C). Particularly, intrinsic MCF strongly correlated with Clauss fibrinogen in both healthy volunteers (*r* = 0.64) and liver transplant patients (*r* = 0.75). Even stronger correlation was achieved between intrinsic MFL and fibrinogen concentration (*r* = 0.81 and 0.88, respectively). Additionally, a strong correlation (*r* ≥ 0.63) was observed between extrinsic MCF or MFL and Clauss fibrinogen (Supplementary Figure S4B).

### 3.5 | i-QATT accurately assesses the reversal of heparin anticoagulation

**FIGURE 7:**
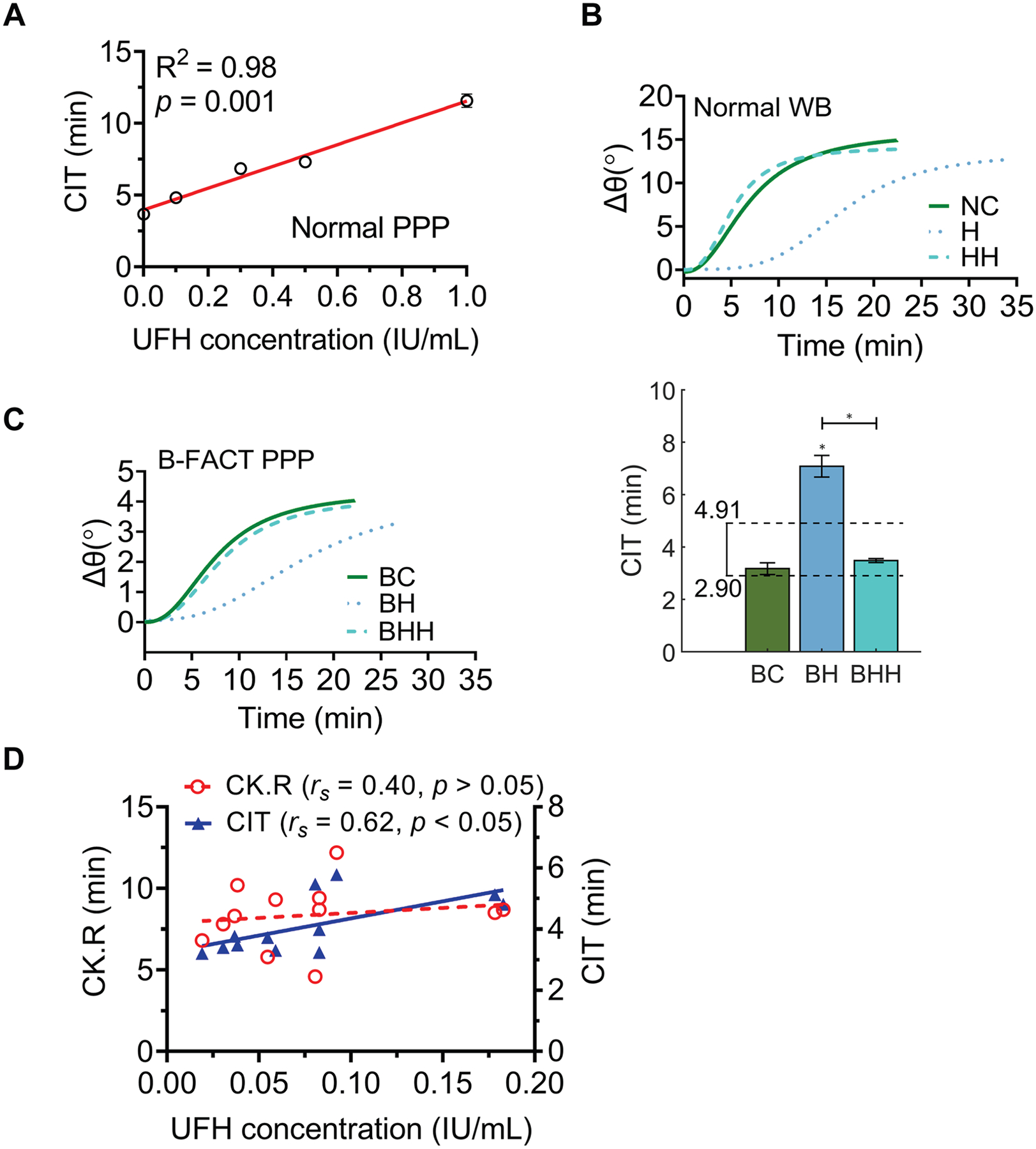
i-QATT measurements of heparin anticoagulation and its reversal in PPP and WB samples activated via the intrinsic pathway. (A) UFH dose response measured by i-QATT CIT in normal PPP samples (*n* = 3 per concentration). (B) Mechanical tweezographs of WB samples from healthy volunteers treated with UFH at a final concentration of 0.5 IU/mL (H), treated with UFH followed by heparinase at a final concentration of 0.11 IU/μL (HH), or left untreated (NC). *n* = 3. (C) Mechanical tweezographs (left) and CIT values (right) of B-FACT PPP treated with UFH at a concentration of 0.4 IU/mL (BH), treated with UFH followed by 0.11 IU/μL heparinase (BHH), or left untreated (BC); *n* = 3. (D) Correlation plots of i-QATT CIT vs TEG-assessed UFH concentration and TEG CK.R vs. TEG-assessed UFH concentration in liver transplant WB samples collected after the arterial reperfusion and during the biliary reconstruction (*n* = 12). CIT, clot initiation time; CK, citrated kaolin; i-QATT, integrated quasistatic acoustic tweezing thromboelastometry; NC, normal control; PPP, platelet-poor plasma; TEG, thromboelastography; UFH, unfractionated heparin; WB, whole blood.

UFH is a common nonoral anticoagulant used in surgeries, hemodialysis, and extracorporeal membrane oxygenation [37–39]. As seen in Figure 7A, CIT exhibited a strong linear relationship with UFH concentration ranging from 0 to 1 IU/mL, a range encompassing doses used in liver transplant procedures, in intrinsic pathway–activated PPP samples (*R*^2^ = 0.98). Other time parameters (TFCF and CT) also linearly changed with UFH concentration (*R*^2^ ≥ 0.94) but yielded indeterminate values at ≥0.5 IU/mL (Supplementary Figure S5A).

Low-dose UFH is reversible by heparinase [40]. The reversal is clearly seen in mechanical tweezographs and CIT values of heparinized WB (H) and B-FACT PPP (BH) samples activated via the intrinsic pathway (Figure 7B, C and first plot in Figure 3C). Heparinase treatment (HH) reduced WB CIT by 72% ± 0.46% compared with heparinized samples (H; *P* < .05), restoring CIT to values comparable with those of untreated controls (5.5% ± 2.0% difference; *P* > .05) and within the reference range (first plot in Figure 3C). Similarly, heparinase reduced CIT in heparinized B-FACT samples (BH) by 50.6% ± 2.15% (*P* < .05), with no significant difference between heparinase-treated samples (BHH) and untreated controls (BC; 14.3% ± 7.97%; *P* > .05) (Figure 7C, right).

Liver transplant patients received UFH therapy after the baseline time point; thus, blood samples collected at the artery and final time points were heparinized. To measure the actual plasma concentration of UFH in those samples, referred to as TEG-assessed UFH concentration, we calculated the ratio of TEG R values between the CK and CKH channels (CK.R/CKH.R) and used the previously established relationship between CK.R/CKH.R and UFH concentration [41]. Figure 7D shows correlation plots of TEG R and i-QATT CIT vs TEG-assessed UFH concentration for liver transplant WB samples collected at the artery and final time points. While R exhibited only a moderate and nonsignificant correlation with UFH concentration (*r* = 0.40), CIT demonstrated a substantially stronger and statistically significant (*r* = 0.62; *P* < .05) correlation.

Further results, including Supplementary Figures S6–S11 and Tables S1–S3, are provided in the Supplementary Information and discussed in its Supplementary Results.

## 4 | DISCUSSION

This study demonstrates that i-QATT rapidly, within 3 to 8 minutes, identifies coagulation defects in PPP and WB samples, surpassing traditional VHAs like TEG. This rapid assessment supports clinical decision making in time-sensitive settings, including hemostatic resuscitation in trauma patients [42] and transfusion during cardiac/liver surgery [43,44]. It is also critical in life-threatening pathophysiological conditions such as sepsis and liver failure, which are often associated with complex coagulopathies [45,46].

We established and validated the reference ranges for i-QATT coagulation parameters. Early-stage parameters (CIT and RT) showed low variability in normal PPP and WB samples due to their primary dependence on coagulation factors, which are relatively consistent in healthy individuals. Late-stage parameters (MCF and TFCF) exhibited higher variability, particularly in WB, due to physiological variation in fibrinogen concentration and platelet activity [47].

i-QATT parameters demonstrated sensitivity to coagulation changes induced by single-factor deficiencies, heparin, abnormal fibrinogen levels, and liver transplantation. Single-factor deficiencies were best detected in PPP by the time parameters CIT, TFCF, and FNFT for the intrinsic pathway and RT for the extrinsic pathway.

Intrinsic CIT and RT exhibited the greatest heparin sensitivity among i-QATT parameters, whereas CT and FNFT were most responsive to heparin in the extrinsic pathway–activated PPP. In WB samples, CIT was the most sensitive parameter to heparin anticoagulation in both pathways, consistent with TEG R and ROTEM CT [5,38]. PPP CIT exhibited a strong linear response to heparin concentrations across the 0- to 1-IU/mL range, highlighting i-QATT suitability for monitoring both prophylactic and therapeutic anticoagulation [39,40]. As previously shown, i-QATT can measure even higher heparin doses in WB samples [30].

Our data show that i-QATT effectively measures heparin reversal by heparinase in both PPP and WB. This capability is important to identify whether prolonged coagulation in surgical patients is predominantly caused by residual heparin or unrelated coagulation defects developed during surgery. In a bleeding patient, this information guides decisions on protamine administration (to reverse heparin) or transfusion of blood products such as fresh frozen plasma. Note that the influence of nonheparin factors on coagulation delay can be excluded by analyzing the CIT ratio between heparinase-treated and untreated samples. Similarly, we anticipate that i-QATT may detect direct oral anticoagulants using specific enzyme activators, such as ecarin for thrombin inhibitors (eg, dabigatran) and Russel viper venom for FXa inhibitors (eg, apixaban) [48,49].

Fibrinogen functional levels can be reliably assessed in PPP samples using i-QATT MCF and MFL. In WB samples, the correlation between MCF and Clauss fibrinogen was reduced due to the masking effect of platelets [50]. In this situation, fibrinogen levels can instead be assessed from MCF in CytD-treated WB samples or from CR in untreated samples. In the intrinsic pathway, WB TFCF was also sensitive to fibrinogen. Using CR, we detected abnormally high levels of fibrinogen in liver transplant patients, likely caused by abnormal thrombin generation and associated changes in fibrin structure [51].

The platelet contribution to clot firmness was quantified by MCF_p_, a difference in MCF between untreated and CytD-treated WB samples. The ratio of MCF_p_ to platelet count demonstrated increased activity of platelets in liver transplant patients compared with that in healthy adults. This result is in line with clinical observation of increased platelet activation during liver transplantation [52].

i-QATT shows potential for detecting coagulopathy associated with end-stage liver disease and liver transplantation. Coagulopathy is primarily characterized by acquired multifactor deficiency developed as a result of the depletion of coagulation factors, which often occurs in end-stage liver disease [53]. In PPP samples, MFL showed relatively high sensitivity and specificity to liver transplantation in both intrinsic and extrinsic pathways, and extrinsic RT (which is equivalent to PT/INR) emerged as the most reliable indicator. Note that PT/INR is more widely accepted than APTT for assessing liver dysfunction, and it is also a key component of the model for end-stage liver disease, a scoring system used to evaluate disease severity and prioritize liver transplantation [54]. In WB samples, CT and CR appeared most responsive to coagulation changes in liver transplant patients for intrinsic– and extrinsic pathway–activated samples, respectively. When focusing on coagulopathic patients with a high bleeding risk (INR ≥ 1.5 and platelet count < 100 000/μL) [55], extrinsic CIT showed the highest responsiveness among i-QATT parameters.

The consistently high correlations observed between i-QATT and gold standard coagulation assays such as INR, APTT, Clauss fibrinogen, and TEG underscore the clinical relevance and diagnostic potential of the technique. Using only a fraction of the sample volume required by conventional assays, i-QATT provides a comprehensive assessment of blood coagulation—functionality that typically requires multiple separate devices.

This study has several limitations. First, i-QATT reference ranges were established using a relatively small sample set. Correlation analyses between i-QATT and gold standard coagulation assays, particularly TEG, would also benefit from a larger sample size for a more robust analytical agreement assessment. Second, although i-QATT is designed to operate using small blood volumes, this study used venous blood collected by venipuncture into standard blood collection tubes to ensure consistency with existing assays. Future work will explore small-volume collection systems and finger-prick capillary blood samples. In terms of technical constraints, the i-QATT photo-optical output, including FFR and MFL, is affected by initial sample darkness (eg, due to hemolysis, bilirubin, or lipids) [56]. In contrast, mechanical measurements, referred to as QATT, remain robust in both WB and PPP samples and should therefore be considered the primary i-QATT readout, with photo-optical measurements serving as complementary information when nondark plasma samples are analyzed. At present, the i-QATT device supports a single assay at a time; however, development is underway to enable simultaneous multiassay operation.

This study demonstrates the clinical potential of i-QATT as a rapid, low-volume, containerless platform for sensitive coagulation analysis that combines features of conventional plasma-based assays with viscoelastic hemostatic measurements. This technique can evaluate hemostatic function including platelet activity in WB using its mechanical readout (QATT). This is valuable for time-sensitive point-of-care management of transfusion, coagulopathy, and anti-platelet/anticoagulant therapies. For a more comprehensive analysis, including the effects of coagulation factors, i-QATT can be performed on PPP samples. The combined mechanical and photo-optical readouts of plasma i-QATT are particularly useful for screening and management of congenital and acquired factor deficiencies, potentially alongside chromogenic assays. Importantly, the reduced blood volume required by our technique minimizes reagent use, leading to lower per-test consumable costs. Our future studies will evaluate the generalizability of i-QATT across broader clinical applications, including anticoagulant therapy monitoring, management of coagulation disorders, and transfusion guidance, and will further optimize the technique for routine point-of-care implementation.

## Supplementary Material

1

The online version contains supplementary material available at https://doi.org/10.1016/j.jtha.2025.12.020.

## Figures and Tables

**TABLE:** Reference ranges for i-QATT coagulation parameters in PPP and WB samples activated via intrinsic and extrinsic pathways.

Parameters	Reference range	90% CI interval of lower limit	90% CI interval of upper limit	Sample size (*n*)
PPP				
Intrinsic QATT				
CIT (min)	2.90–4.91	2.68–3.14	4.66–5.15	33
TFCF (min)	4.71–7.84	4.54–4.93	7.10–8.75	34
CT (min)	1.13–3.06	1.01–1.27	2.68–3.51	33
CR (°/min)	1.92–10.59	1.01–3.00	9.51–11.67	34
MCF (°)	4.08–10.70	3.35–4.86	9.88–11.48	33
RT (min)	2.58–4.39	2.36–2.81	4.16–4.61	33
MFL (gs)	15.94–38.48	13.49–18.55	35.74–41.11	34
FFR (gs/min)	11.98–37.39	8.81–15.15	34.17–40.35	34
FNFT (min)	1.50–3.05	1.33–1.68	2.86–3.24	33
Extrinsic QATT				
CIT (min)	0.64–1.97	0.49–0.80	1.80–2.13	32
TFCF (min)	2.30–5.95	1.84–2.78	5.52–6.40	34
CT (min)	1.38–4.21	1.06–1.71	3.86–4.56	32
CR (°/min)	1.75–8.45	0.93–2.55	7.60–9.28	34
MCF (°)	5.31–12.51	4.52–6.16	11.62–13.36	33
RT (min)	0.13–0.87	0.04–0.22	0.77–0.96	32
MFL (gs)	15.39–38.97	12.83–18.12	36.10–41.72	34
FFR (gs/min)	6.96–24.56	4.58–9.16	22.45–26.51	34
FNFT (min)	2.01–5.20	1.65–2.39	4.80–5.58	32
WB				
Intrinsic QATT				
CIT (min)	1.09–4.12	0.75–1.44	3.74–4.48	33
TFCF (min)	8.95–21.07	7.55–10.44	19.50–22.58	30
CT (min)	7.42–19.94	6.57–8.41	17.52–22.78	30
CR (°/min)	0.56–3.00	0.48–0.67	2.38–3.69	30
MCF (°)	4.57–19.61	2.83–6.42	17.66–21.47	30
Extrinsic QATT				
CIT (min)	0.63–2.47	0.56–0.74	2.06–2.93	34
TFCF (min)	4.03–19.55	2.24–5.95	17.54–21.48	30
CT (min)	2.98–18.07	1.23–4.84	16.12–19.95	30
CR (°/min)	0.15–3.31	0–0.54	2.90–3.70	30
MCF (°)	4.59–20.86	2.71–6.59	18.75–22.88	30
CytD				
Intrinsic QATT–MCF (°)	1.66–7.30	1.02–2.34	6.58–7.99	31
Extrinsic QATT–MCF (°)	2.45–9.09	1.50–3.48	8.01–10.13	19

CIT, clot initiation time; CR, clotting rate; CT, clotting time; CyT, cytochalasin-D; FFR, fibrin formation rate; FNFT, fibrin network formation time; MCF, maximum clot firmness; MFL, maximum fibrin level; i-QATT, integrated quasistatic acoustic tweezing thromboelastometry; PPP, platelet-poor plasma; RT, reaction time; TFCF, time to firm clot formation; WB, whole blood.

## Essentials

- Large sample volumes limit coagulation testing and risk iatrogenic anemia in vulnerable patients.
- i-QATT is a novel containerless method for coagulation analysis that requires only 6 μL of blood.
- i-QATT rapidly detects coagulation defects and correlates strongly with gold-standard assays.
- It is sensitive to factor deficiencies, anticoagulation, coagulopathy and can assess fibrinogen and platelet activity.
