# Supplementary material for: Establishing the clinical utility of integrated quasistatic acoustic tweezing thromboelastometry for blood coagulation analysis
Source: J Thromb Haemost. Author manuscript; Available in PMC 2026 Jul 2. (PMC13326700; doi:10.1016/j.jtha.2025.12.020)
Supplement: 1 [file NIHMS2189471-supplement-1.pdf]

Supplementary Information

“Establishing the clinical utility of integrated quasi-static acoustic tweezing thromboelastometry for blood coagulation analysis”

by

Huy Q. Pham, Collette Barnor, Trishita Paul, Elizabeth M. Cummins, Daniel Arango, Shaun Yockelson, Damir B. Khismatullin

Supplementary Methods

Commercial blood samples

Platelet-poor plasmas deficient (PPP) in fibrinogen (Fg) and FII, FV, FVIII, FXI, and FXII were purchased from Affinity Biologicals (Ontario, Canada). FXIII-deficient PPP was obtained from George King Bio-Medical (Overland Park, KS). Normal coagulation control (Level 1) PPP was purchased from Thermo Fisher Scientific (Waltham, MA).

Specificity/sensitivity analyses

The diagnostic performance of i-QATT^{TM} parameters was evaluated through sensitivity and specificity analyses. A test result (within-range or outside-range) was assigned to each sample by comparing its parameter value to the corresponding reference range. The actual condition of each sample (normal or abnormal) was defined based on clinical grouping (e.g., samples from healthy volunteers labeled as normal; samples from liver transplant patients, heparinized samples, and samples with low or high fibrinogen levels labeled as abnormal). The test outcome categories, including true positives (TP), true negatives (TN), false positives (FP), and false negatives (FN), were determined by comparing the test result with the actual condition. Sensitivity was calculated as TP / (TP + FN), and specificity as TN / (TN + FP).

Assessment of i-QATT^{TM} repeatability measurements

We assessed the repeatability of i-QATT^{TM} measurements using the coefficient of variation (CV), calculated from experiments involving three independently prepared aliquots of the same plasma sample, with three drops tested from each aliquot. CV was calculated for each aliquot (Supplementary Table S1a) and three aliquots (Supplementary Table S1b) as the ratio of standard deviation s to the mean $\bar{x}$, expressed as a percentage:

$$\mathrm{CV}\ (\%) = \left(\frac{s}{\bar{x}}\right) \times 100 \tag{1}$$

The coefficient of biological variation (BV) was calculated from the healthy volunteer dataset using Eq. (1), where s represents the standard deviation and $\bar{x}$ denotes the mean of the dataset.

Supplementary Results

i-QATT^{TM} shows acceptable precision, with most CVs below 10%

The precision data (obtained from tweezographs shown in Supplementary Fig. S6), including repeatability (within-day measurements) and intermediate precision (between-day measurements), are summarized in Supplementary Table S1a. Both CIT and TFCF had very low CVs varied between 1.0 to 4.8% for different aliquots. The CV increased to about 10% for most of the other parameters with the highest values less than 19.0%. These CVs fall within the generally acceptable range for intra-laboratory precision [1]. The intermediate precision ranged from 1.5 to 9.5% for CIT, RT, TFCF, CT, MCF, and MFL, but it was 10% or more for FNFT and the rate parameters, CR and FFR. As shown in Supplementary Table S1b, the biological variation (BV) calculated from PPP data across all healthy subjects were higher than CV derived from nine technical replicates of a single PPP sample (three aliquots × three drops), indicating a substantial contribution of biological variability to the i-QATT^{TM} measurements. Note that the 9-drop CV for i-QATT^{TM} CIT was 3.680%, which was less than CVs reported for TEG R-time (10-16%) and ROTEM CT (4-5%). However, the CV for i-QATT^{TM} MCF (9.066%) was higher than TEG MA CVs (4-7%) and ROTEM MCF CVs (1.2-2.6%) [2-4].

i-QATT™ shows strong potential for discerning normal from abnormal coagulation

To assess the diagnostic performance of i-QATT™, we evaluated the sensitivity and specificity of its parameters across various clinically relevant conditions. These include UFH anticoagulation, low and high fibrinogen levels, single factor deficiencies, and liver transplantation. This analysis, shown in Supplementary Tables S2 and S3, highlights the capability of individual parameters to detect specific abnormalities under both intrinsic and extrinsic pathway activation.

In plasma, both CIT and RT had high sensitivity (83% and 89%) and specificity (94% and 97%, Supplementary Table S2a) to UFH-treated samples activated via the intrinsic pathway. For the extrinsic pathway, PPP CT and FNFT had the highest sensitivity (89%) and specificity (100% and 97%) among all parameters. For fibrinogen level assessment, PPP MCF was most sensitive (>83%) and specific (>88%) for both pathways (Supplementary Table S2b). For factor deficiency detection (tweezographs shown in Supplementary Fig. S7) , FNFT, CIT, and TFCF all had high sensitivity (92%, 84%, 81%) and specificity (97%, 94%, 97%) for intrinsic pathway-activated samples, while extrinsic RT had relatively good sensitivity (65%) and high specificity (97%, Supplementary Table S2c). Since liver transplant patients represent a clinical group with a heterogeneous coagulation profile, we did not expect high sensitivity of i-QATT parameters to this condition. Nevertheless, we found that MFL was reasonably sensitive to liver transplantation in both intrinsic and extrinsic activated PPP (62% and 86%, Supplementary Table S2d). Also, we obtained the highest sensitivity (100%) of PPP RT in the extrinsic pathway.

In whole blood samples, UFH anticoagulation was best detected by CIT, which demonstrated the highest sensitivity (100%) and specificity (>97%) across both pathways (Supplementary Table S3a). TFCF also had 100% sensitivity to UFH. While CT was not sensitive to UFH in the intrinsic pathway, abnormally high values of TFCF in the extrinsic pathway led to 100% sensitivity of this parameter. For changes in fibrinogen level, CR was the most sensitive parameter (90% in the intrinsic pathway and 100% in the extrinsic one; see correlation plots of CR and Clauss fibrinogen - Supplementary Figure S8a), while CT and TFCF also demonstrated the highest sensitivity (100%) in the intrinsic pathway (Supplementary Table S3b). For evaluating liver transplant patients, CT and CR were most sensitive (57% and 65%) to this condition in intrinsic and extrinsic pathway activated WB samples, respectively (Supplementary Table S3c). To evaluate the sensitivity of i-QATT™ to coagulopathic patients with a high bleeding risk (INR ≥ 1.5 and platelet count < 100,000/µL), we classified liver transplant WB samples with INR < 1.5 and platelet counts ≥ 100,000/µL as non-coagulopathic and included them in the normal group. Based on this classification, CIT demonstrated a sensitivity of 60% for detecting coagulopathy in extrinsic pathway activated WB (Supplementary Table S3d).

i-QATT™ is sensitive to fibrinogen concentration and platelet activity

Like MCF, CR depends on both fibrinogen concentration and platelet activity. When comparing WB data from healthy and liver transplant subjects, there was a highly significant increase in the ratios of CR to fibrinogen and to platelet count in the liver transplant group (Supplementary Fig. S8b,c). This accounts for the heightened sensitivity of CR in liver transplant patients, as both fibrinogen and platelet count exert a much stronger influence on this parameter. The scatter plots in Supplementary Fig. S8b,c show that a change in CR with fibrinogen or platelet count is small in healthy subjects but this change becomes very large in liver transplant subjects.

i-QATT™ accurately measures heparin reversal and can differentiate heparin effects from those of factor deficiency or non-vitamin K antagonists.

Compared to intrinsic pathway-activated blood samples, similar (and much stronger) effects of UFH and heparinase were observed in extrinsic pathway-activated WB (Supplementary Fig. S5b and first plot in Fig. 3d), but the UFH dose effect is less pronounced for this pathway based on the PPP data as we discussed before (first plot in Fig. 3b).

As shown in Supplementary Fig. S9, heparinase effectively reversed the prolonged coagulation caused by heparin but not that induced by FVII-deficiency or apixaban (a vitamin K antagonist). Only heparinized PPP returned to normal clotting after heparinase treatment (Supplementary Fig. S9a,b). Consequently, the ratio of CIT before to after heparinase treatment was much higher for heparinized PPP (4.09) than for FVII-deficient (1.25) and apixaban-spiked PPP (1.14).

i-QATT^{TM} detects coagulation changes across different time points during liver transplantation

The i-QATT^{TM} graphical outputs for PPP and WB samples from liver transplant patients (representative and average data) collected at different time points during surgeries are summarized in Supplementary Figs. S10 and S11, respectively. The data revealed three main scenarios for surgery-associated coagulation changes. In one patient, clot firmness was much higher at the baseline time point than at later points while no changes were observed for coagulation time (first column in Supplementary Figs. S10 and S11). This suggests that this patient may have the hypercoagulable state before surgery, detected by abnormally low TFCF and high CR values (Supplementary Fig. S11c, first and second plots). In another patient, coagulation in PPP samples was prolonged and clot firmness was small at the baseline and artery time points compared to the final point. This pattern indicates a hypocoagulable state before and during surgery, with normalization of hemostatic function after transplantation, likely due to intraoperative blood product administration (second column in Supplementary Fig. S10). However, WB samples in this case showed normal behavior at the baseline and final points and delayed coagulation at the artery point, which may indicate the compensatory effect of platelets prior to surgery and the lack of this effect during the arterial reperfusion (second column in Supplementary Fig. S11). This patient had abnormally high WB CIT value at the artery point (Supplementary Fig. S11c, third plot). The third scenario is indicative of rebalanced hemostasis, in which PPP samples had prolonged coagulation and reduced clot firmness at the baseline but not at the artery and final points; however, WB samples demonstrated normal coagulation in the intrinsic pathway at all points (third column in Supplementary Figs. S10 and S11). The extrinsic pathway WB data showed normal coagulation with reduced clot firmness at the baseline (Supplementary Fig. S11c, fourth plot), consistent with ROTEM EXTEM findings on rebalanced hemostasis in liver disease patients [5-7].

Supplementary Tables and Figures

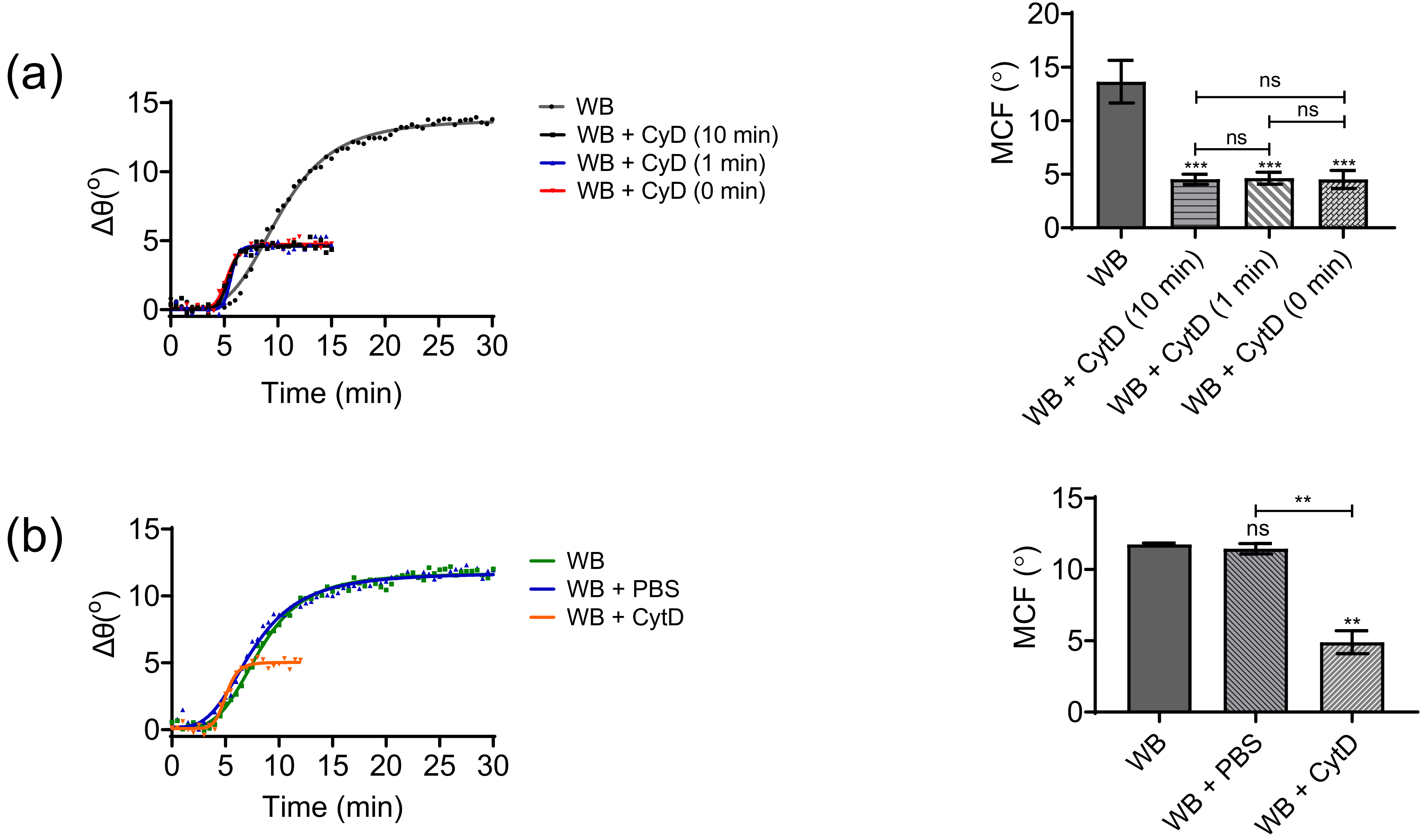

Supplementary Figure S1. (a) Cytochalasin-D effect on whole blood with different incubation time (0, 1, 10). (b) Comparison of Cytochalasin-D (first dissolved in DMSO and then diluted in PBS) with a PBS-only control.

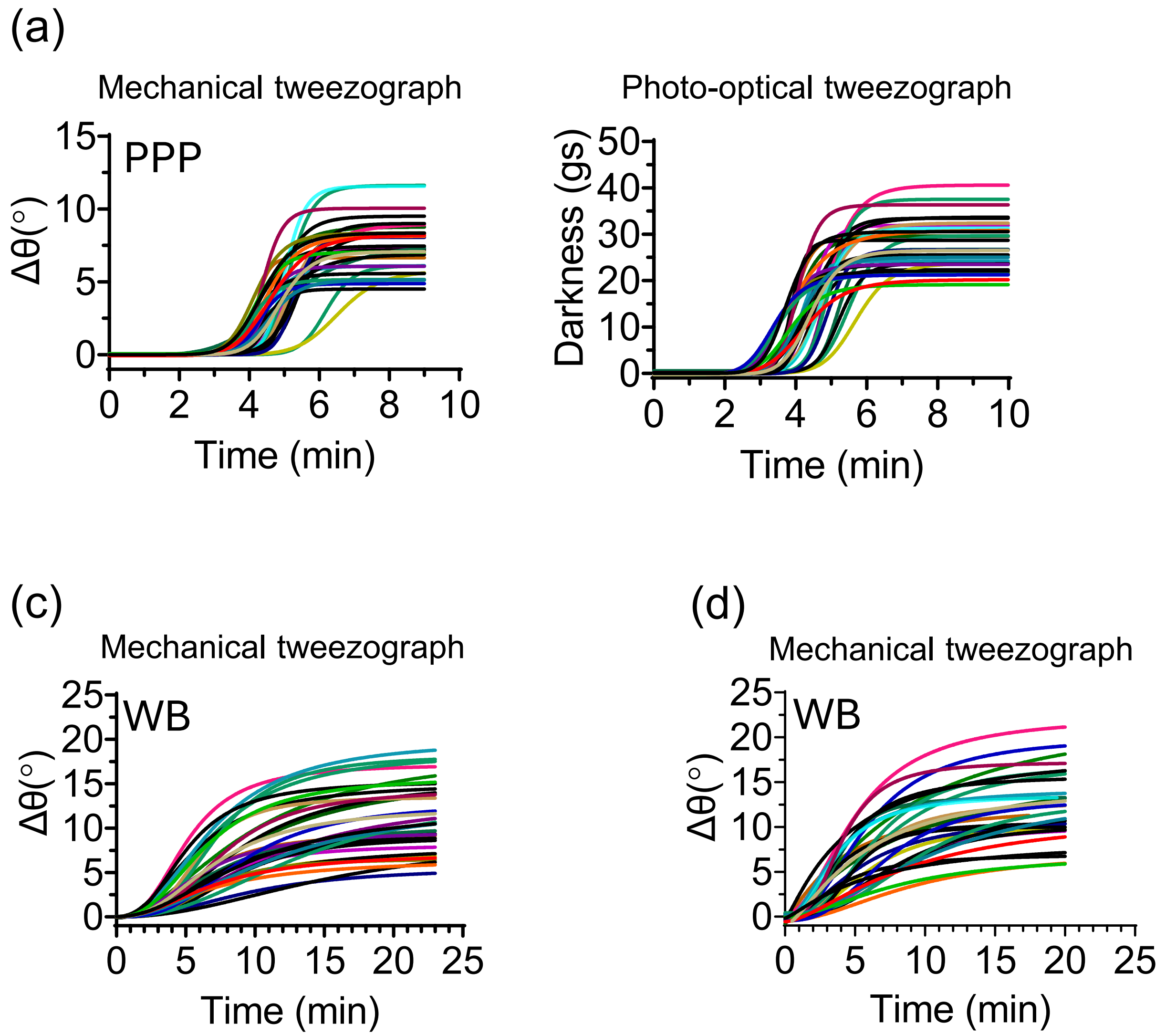

Supplementary Figure S2. Mechanical and photo-optical tweezographs of PPP samples and mechanical tweezographs of WB samples from healthy volunteers. (a,b) PPP samples activated via the intrinsic (a) or extrinsic (b) pathway. (c,d) WB samples activated via the intrinsic (c) or extrinsic (d) pathway. Sample size: n = 30 to 33.

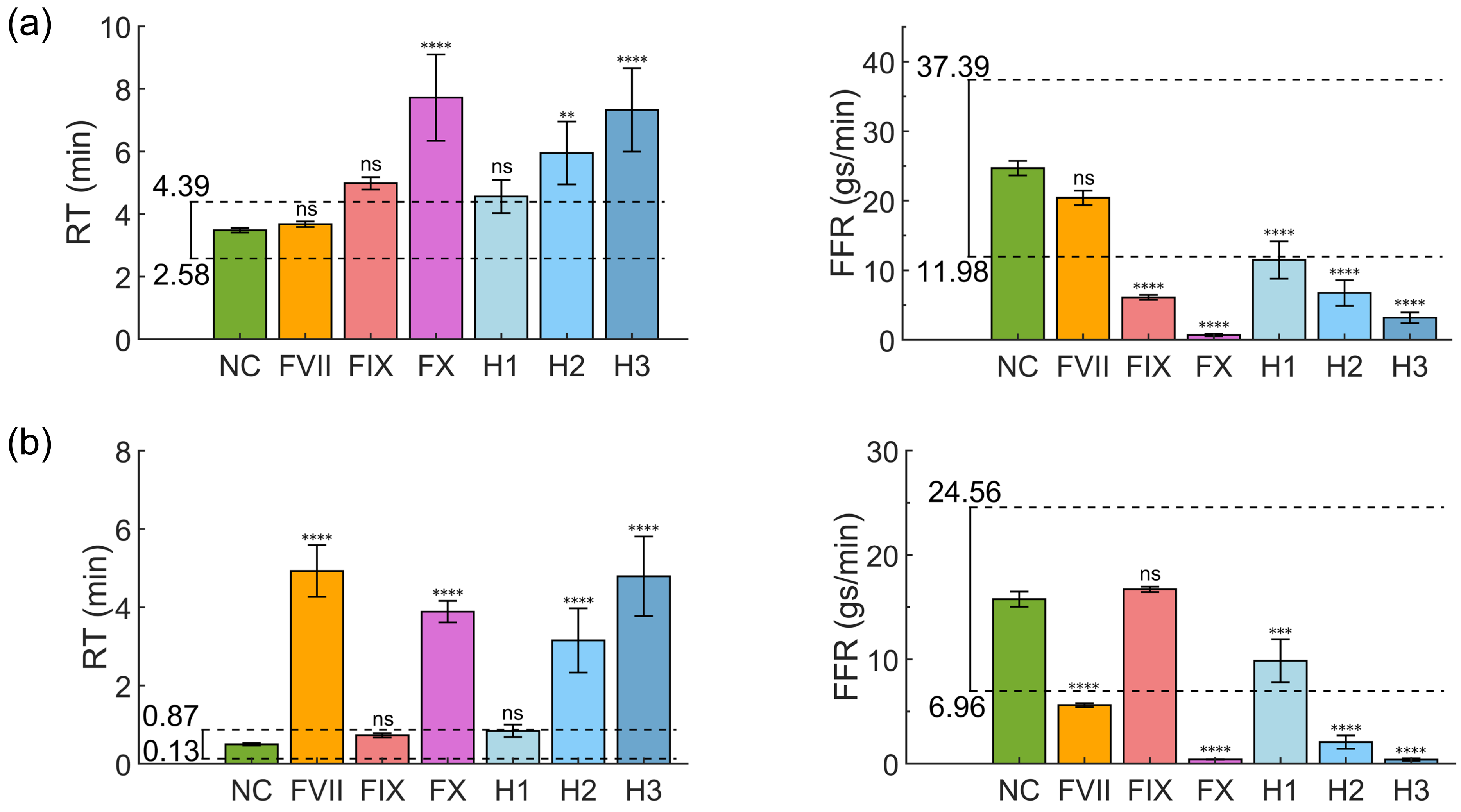

Supplementary Figure S3. Validation of i-QATT™ reference ranges for photo-optical parameters in platelet-poor plasma (PPP) samples activated via the intrinsic (a) and extrinsic (b) pathways. *$p < 0.05$, **$p < 0.01$, ***$p < 0.001$, ****$p < 0.0001$ (n = 6 to 33-34 for intrinsic pathway and n = 6 to 32-34 for extrinsic pathway)

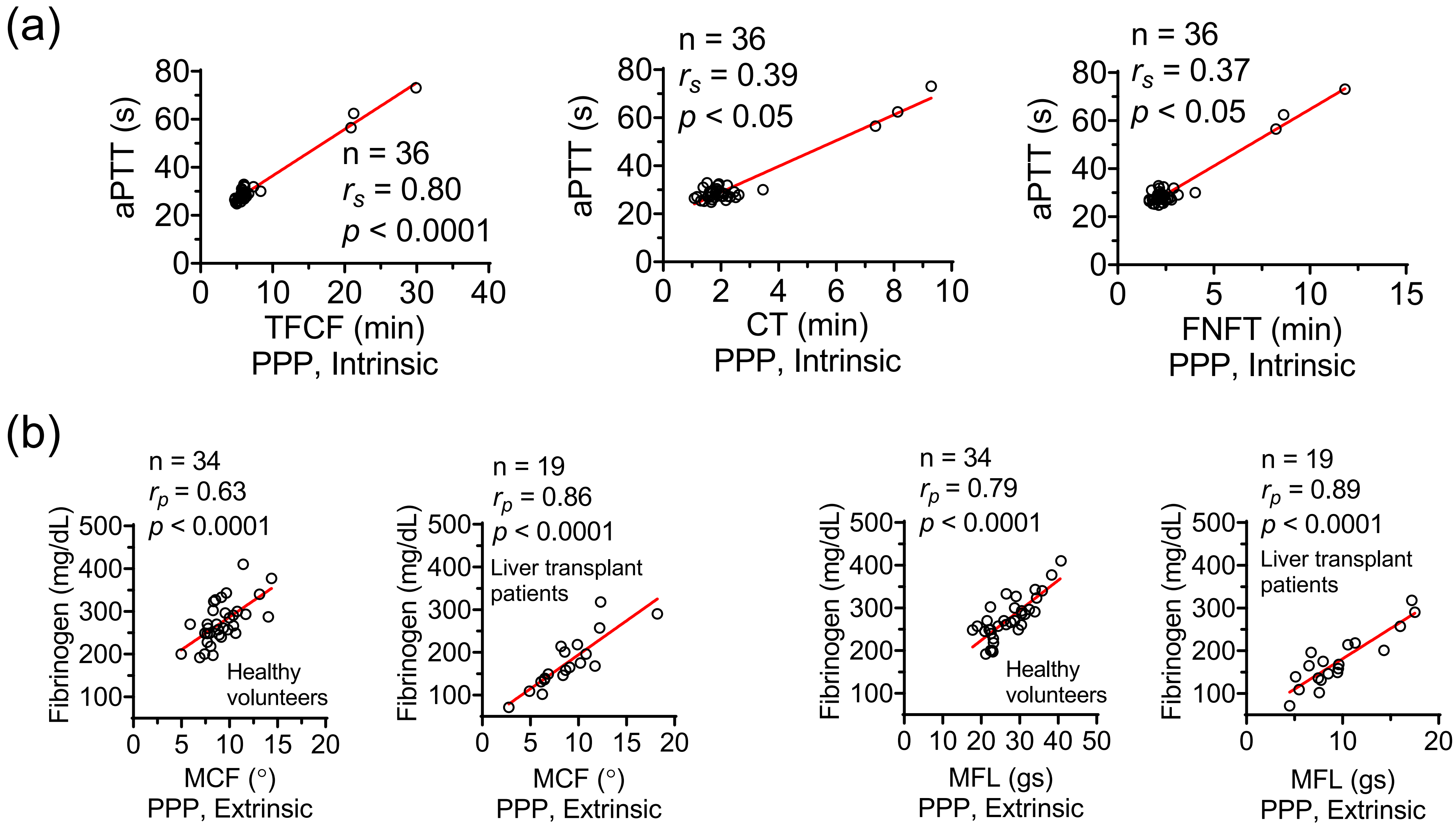

Supplementary Figure S4. (a) Correlation plots of i-QATT^{TM} CT, FNFT, and TFCF vs. aPTT for normal and abnormal PPP samples activated via the intrinsic pathway. (b) i-QATT^{TM} MCF and MFL vs. Clauss fibrinogen for PPP samples from healthy volunteers and liver transplant patients activated via the extrinsic pathway.

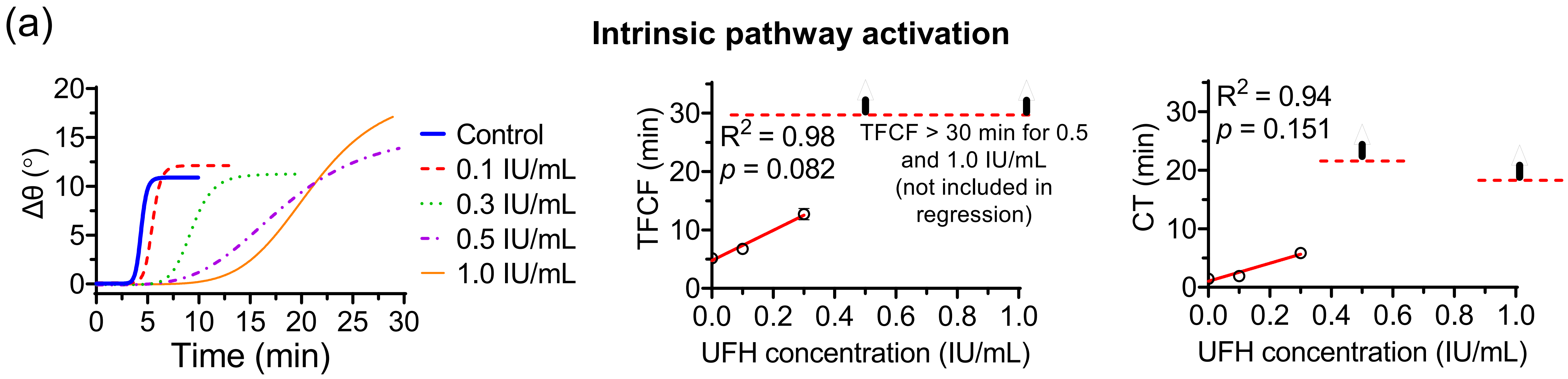

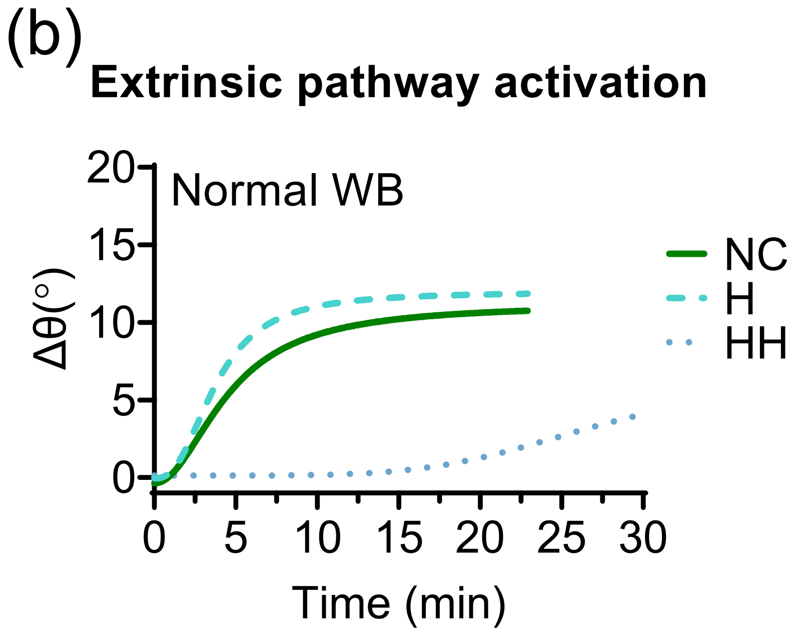

Supplementary Figure S5. (a) Mechanical tweezograph of commercial pooled normal plasmas activated via the intrinsic pathway and UFH dose-response curves of TFCF and CT. ed dashed lines indicate the boundaries of the studied range. (b) Mechanical tweezograph of extrinsic pathway-activated normal whole blood (WB) under three conditions: untreated (NC), heparinized (H), and heparinase-treated following heparinization (HH). n=3.

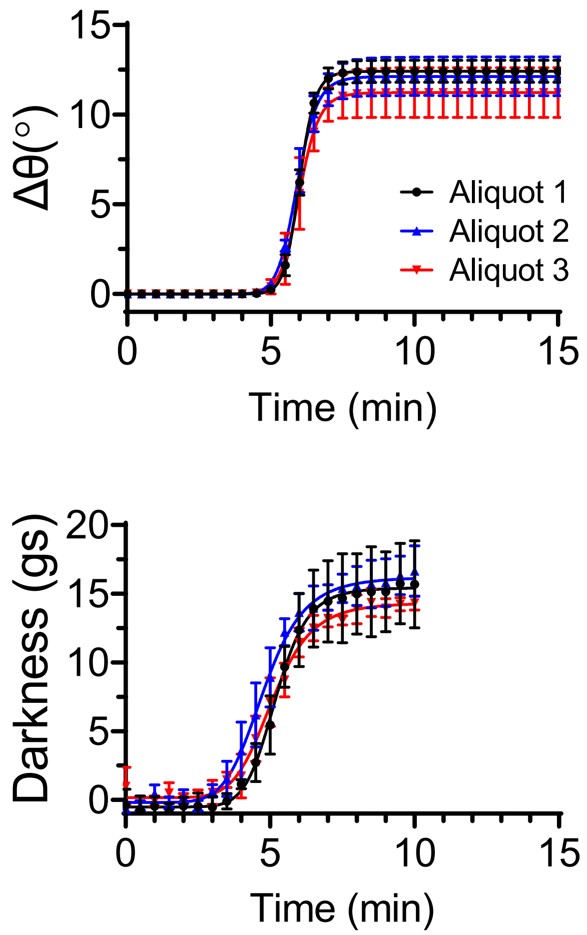

Supplementary Figure S6. Repeatability assessment of i-QATT™ measurements in normal control PPP. (a) Mechanical (top) and photo-optical (bottom) tweezographs of three PPP samples (black, blue, red) measured in triplicate (3 drops per sample). Shown is Mean ± SD.

(a)

Parameter	Aliquot	Replicate 1	Replicate 2	Replicate 3	Mean	SD	CV (%)
CIT	1	5.24	5.36	5.08	5.23	0.14	2.69%
CIT	2	5.00	4.90	4.03	4.64	0.54	1.01%
CIT	3	5.30	5.23	4.84	5.12	0.25	4.84%
TFCF	1	6.76	6.73	6.87	6.79	0.07	1.09%
TFCF	2	6.57	7.20	6.06	6.61	0.57	4.76%
TFCF	3	7.24	6.77	6.67	6.89	0.30	4.41%
CT	1	1.52	1.37	1.79	1.56	0.21	13.64%
CT	2	1.58	2.30	2.04	1.97	0.36	18.48%
CT	3	1.94	1.54	1.83	1.77	0.21	11.68%
CR	1	12.34	12.46	10.00	11.60	1.39	11.97%
CR	2	10.68	7.38	9.18	9.08	1.65	18.20%
CR	3	9.36	9.18	8.93	9.16	0.21	2.34%
MCF	1	13.07	11.83	12.35	12.42	0.62	5.02%
MCF	2	11.33	11.73	13.36	12.14	1.08	8.86%
MCF	3	12.60	9.79	11.30	11.23	1.41	12.52%
RT	1	3.72	3.88	4.38	3.99	0.34	8.63%
RT	2	3.45	3.18	2.73	3.12	0.37	7.89%
RT	3	3.85	3.31	3.26	3.47	0.33	9.40%
FFR	1	6.47	7.12	6.41	6.67	0.40	5.95%
FFR	2	6.87	5.13	6.14	6.05	0.87	14.43%
FFR	3	5.35	4.91	5.09	5.12	0.22	4.30%
MFL	1	17.00	16.20	12.30	15.17	2.52	16.58%
MFL	2	17.40	17.40	14.70	16.50	1.56	9.45%
MFL	3	13.12	15.11	14.17	14.13	1.00	7.04%
FNFT	1	3.05	2.85	2.49	2.80	0.28	10.00%
FNFT	2	3.05	4.02	3.33	3.47	0.50	14.38%
FNFT	3	3.39	3.46	3.41	3.42	0.03	0.99%

(b)

Samples	Healthy volunteers (biological variation)	Single sample (technical variation)
Parameter	BV (%)	CV (%)
CIT	12.46%	3.680%
TFCF	12.94%	3.459%
CT	24.93%	16.71%
CR	33.56%	16.59%
MCF	21.65%	9.066%
RT	12.56%	10.43%
FFR	24.93%	14.05%
MFL	20.07%	12.24%
FNFT	16.48%	13.38%

Supplementary Table S1. Technical and biological variation of i-QATT technique in normal PPP samples. (a) Repeatability assessment using three independently prepared aliquots from the same normal PPP sample, each measured in triplicates. Shown are the individual replicate values along with the calculated mean, standard deviation (SD), and coefficient of variation (CV%). (b) Comparison of biological variation across healthy donor samples (n = 33-34) and technical variation from a single sample (n = 9; three aliquots × three drops) for i-QATT™ parameters.

(a)

Heparin treatment

Parameter	Intrinsic pathway		Extrinsic pathway	
	Sensitivity	Specificity	Sensitivity	Specificity
**CIT**	**83.33%**	**94.12%**	72.22%	94.12%
TFCF	77.78%	97.06%	83.33%	97.06%
**CT**	66.67%	94.12%	**88.89%**	**100.00%**
CR	38.89%	97.06%	66.67%	94.12%
MCF	33.33%	94.12%	27.78%	88.24%
**RT**	**88.89%**	**97.06%**	77.78%	97.06%
FFR	77.78%	100.00%	77.78%	97.06%
MFL	5.56%	97.06%	38.89%	97.06%
**FNFT**	83.33%	97.06%	**88.89%**	**97.06%**

(b)

Fibrinogen

Parameter	Intrinsic pathway		Extrinsic pathway	
	Sensitivity	Specificity	Sensitivity	Specificity
CIT	14.29%	94.12%	16.67%	94.12%
TFCF	42.86%	97.06%	16.67%	97.06%
CT	42.86%	94.12%	16.67%	100.00%
CR	57.14%	97.06%	66.67%	94.12%
**MCF**	**85.71%**	**94.12%**	**83.33%**	**88.24%**
RT	14.29%	97.06%	16.67%	97.06%
FFR	14.29%	100.00%	50.00%	97.06%
MFL	42.86%	97.06%	16.67%	97.06%
FNFT	42.86%	97.06%	16.67%	97.06%

(c)

Factor-deficiency

Parameter	Intrinsic pathway		Extrinsic pathway	
	Sensitivity	Specificity	Sensitivity	Specificity
**CIT**	**83.78%**	**94.12%**	55.88%	94.12%
**TFCF**	**81.08%**	**97.06%**	50.00%	97.06%
CT	72.97%	94.12%	55.88%	100.00%
CR	54.05%	97.06%	44.12%	94.12%
MCF	54.05%	94.12%	44.12%	88.24%
**RT**	72.97%	97.06%	**64.71%**	**97.06%**
FFR	72.97%	100.00%	50.00%	97.06%
MFL	32.43%	97.06%	38.24%	97.06%
**FNFT**	**91.89%**	**97.06%**	52.94%	97.06%

(d)

Liver transplantation

Parameter	Intrinsic pathway		Extrinsic pathway	
	Sensitivity	Specificity	Sensitivity	Specificity
CIT	33.33%	94.12%	76.19%	94.12%
TFCF	42.86%	97.06%	23.81%	97.06%
CT	38.10%	94.12%	71.43%	100.00%
CR	28.57%	97.06%	61.90%	94.12%
MCF	33.33%	94.12%	23.81%	88.24%
**RT**	33.33%	97.06%	**100.00%**	**97.06%**
FFR	52.38%	100.00%	66.67%	97.06%
**MFL**	**61.90%**	**97.06%**	**85.71%**	**97.06%**
FNFT	47.62%	97.06%	61.90%	97.06%

Supplementary Table S2. Sensitivity and specificity analysis of i-QATT™ parameters for PPP samples under various coagulation conditions. (a) Heparin-spiked PPP samples at 0.1, 0.3, and 0.5 IU/mL (abnormal sample size n = 18). (b) Samples with low and high fibrinogen levels (n = 7 for intrinsic; 6 for extrinsic). (c) Factor-deficient plasma samples (Fg- , FII-, FV-, FVII-, FVIII-, FIX-, FX-, FXI-, FXII-, FXIII-, n = 37 for intrinsic; 34 for extrinsic). (d) Samples from liver transplant recipients (n = 21). Number of samples in the healthy group is 34.

(a)

Heparin treatment

	Intrinsic pathway		Extrinsic pathway	
Parameter	Sensitivity	Specificity	Sensitivity	Specificity
**CIT**	**100.00%**	**96.97%**	**100.00%**	**100.00%**
TFCF	100.00%	87.88%	100.00%	96.97%
CT	0.00%	84.85%	100.00%	96.97%
CR	25.00%	90.91%	0.00%	96.97%
MCF	0.00%	93.94%	66.67%	87.88%

(b)

Fibrinogen

	Intrinsic pathway		Extrinsic pathway	
Parameter	Sensitivity	Specificity	Sensitivity	Specificity
CIT	10.00%	96.97%	25.00%	100.00%
TFCF	100.00%	87.88%	50.00%	97.06%
CT	100.00%	84.85%	37.50%	97.06%
**CR**	**90.00%**	**90.91%**	**100.00%**	**97.06%**
MCF	10.00%	93.94%	12.50%	88.24%

(c)

Liver transplantation

	Intrinsic pathway		Extrinsic pathway	
Parameter	Sensitivity	Specificity	Sensitivity	Specificity
CIT	14.29%	96.97%	30.00%	100.00%
TFCF	52.38%	87.88%	40.00%	97.06%
**CT**	**57.14%**	**84.85%**	35.00%	97.06%
**CR**	23.81%	90.91%	**65.00%**	**97.06%**
MCF	9.52%	93.94%	10.00%	88.24%

(d)

Coagulopathy Associated with Liver Transplantation

	Extrinsic pathway	
Parameter	Sensitivity	Specificity
**CIT**	**60.00%**	**93.75%**
TFCF	60.00%	87.50%
CT	40.00%	87.50%
CR	60.00%	77.08%
MCF	0.00%	89.58%

Supplementary Table S3. Sensitivity and specificity analysis of i-QATT™ parameters for WB samples under various coagulation conditions. (a) Heparin-spiked samples at 0.5 IU/mL (n = 4 for intrinsic; 3 for extrinsic). (b) Samples with high fibrinogen (n = 10 for intrinsic; 8 for extrinsic). (c) Samples from liver transplant recipients (n = 21 for intrinsic; 20 for extrinsic). (d) Samples from liver transplant recipients and suspected to have coagulopathy identified as INR ≥ 1.5 and platelet count < 100,000 (n = 20). Number of samples in the healthy group is 34.

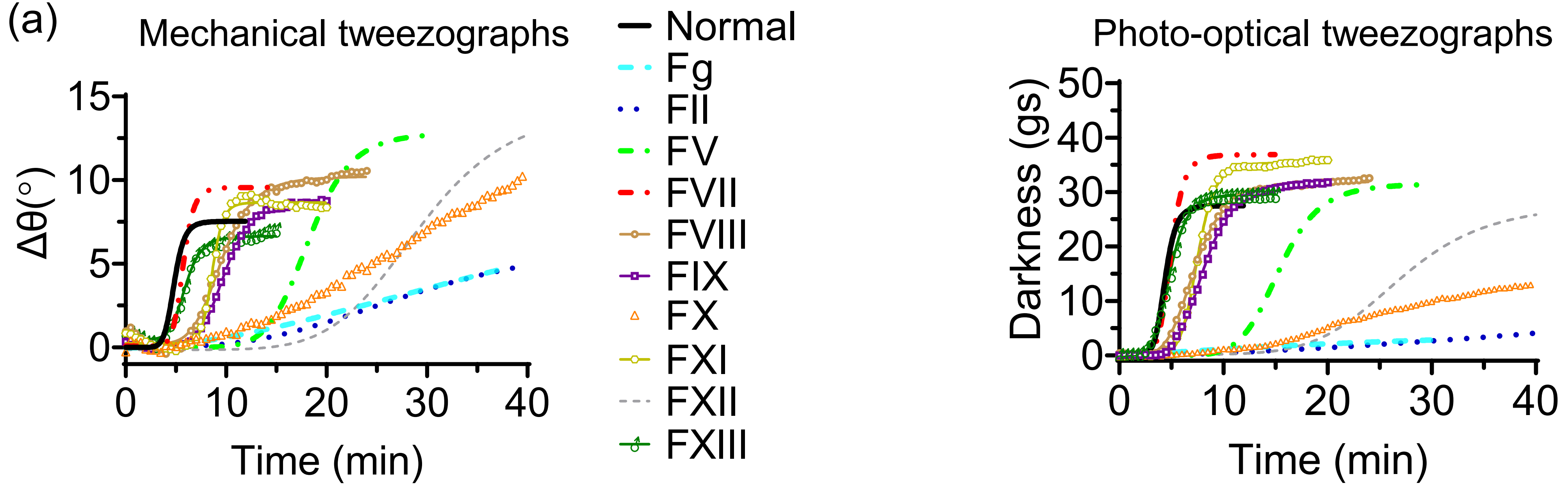

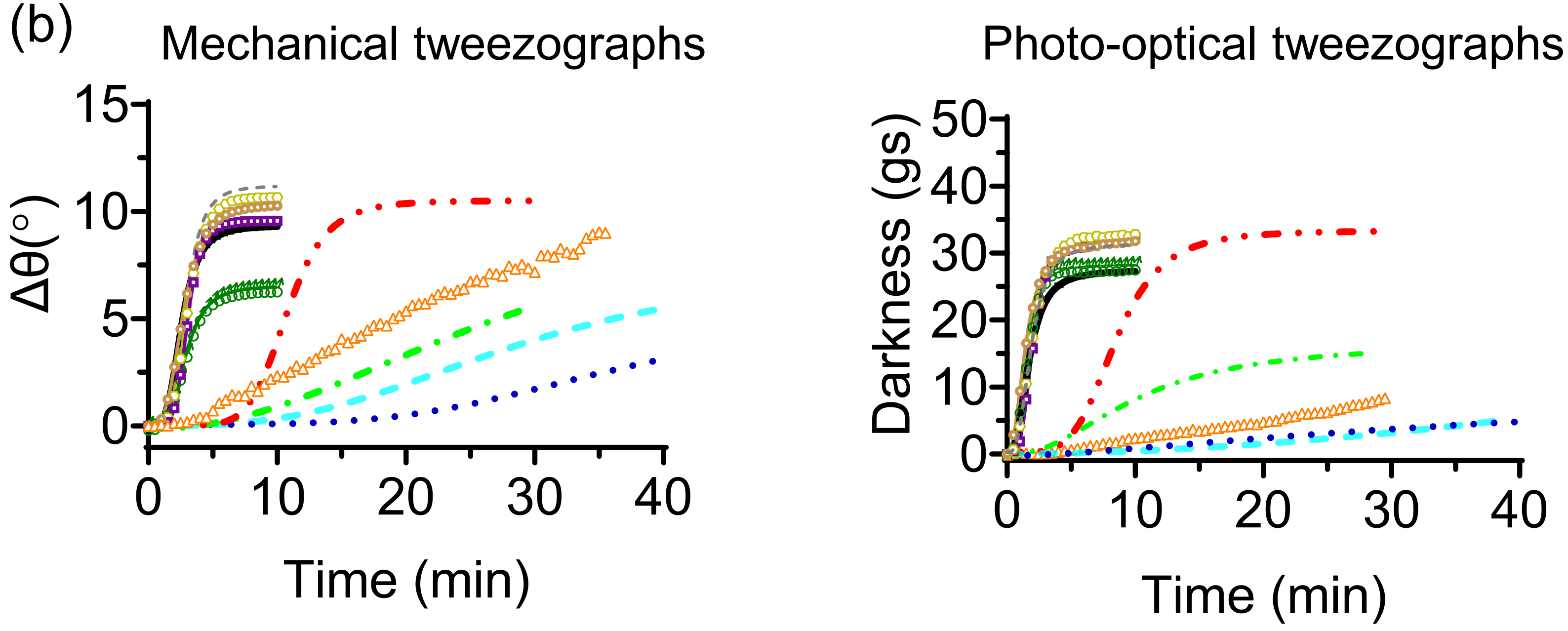

Supplementary Figure S7. Mechanical and photo-optical tweezographs of normal and factor-deficient PPP samples activated by (a) intrinsic and (b) extrinsic pathways. Sample size: n = 3 to 4 independent experiments.

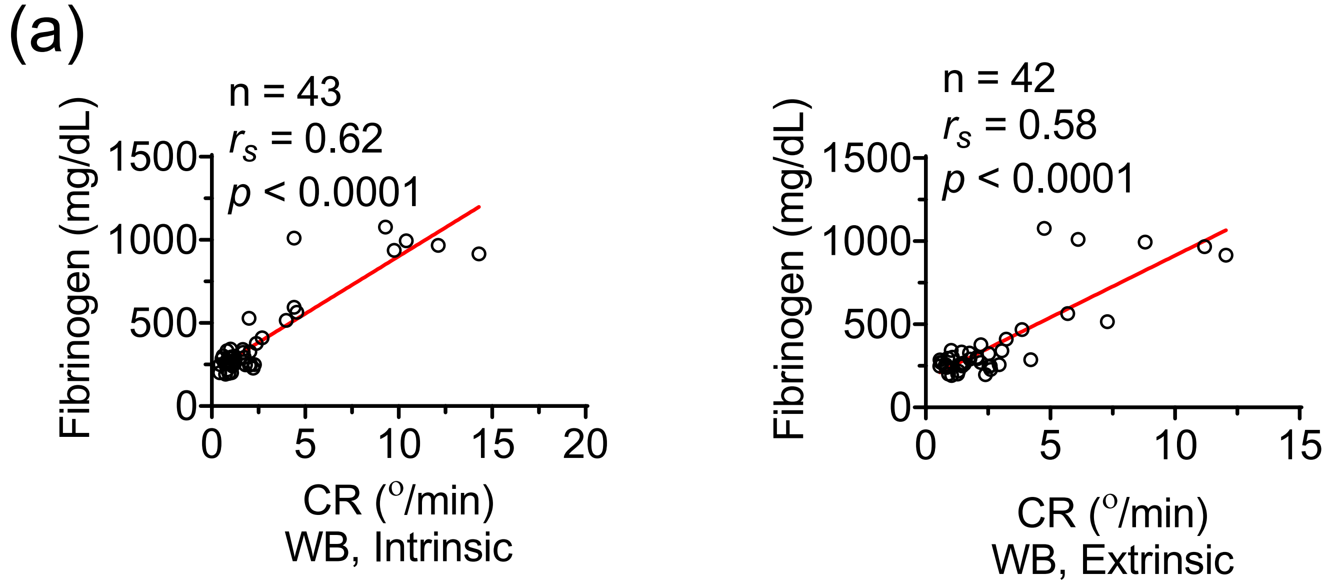

Supplementary Figure S8. (a) Correlation plots of CR vs. fibrinogen concentration in normal WB samples exposed or not to additional fibrinogen, with coagulation activated via the intrinsic (left) and extrinsic (right) pathways. (b-c) Sensitivity of CR to fibrinogen concentration (b) and platelet count (c) in WB samples from healthy volunteers (black) and liver transplant patients (red). The coagulation was activated via the extrinsic pathway. n=33-34 for the healthy group and 19-20 for the liver transplant group. The bar graphs show mean ± SEM. ****p < 0.0001.

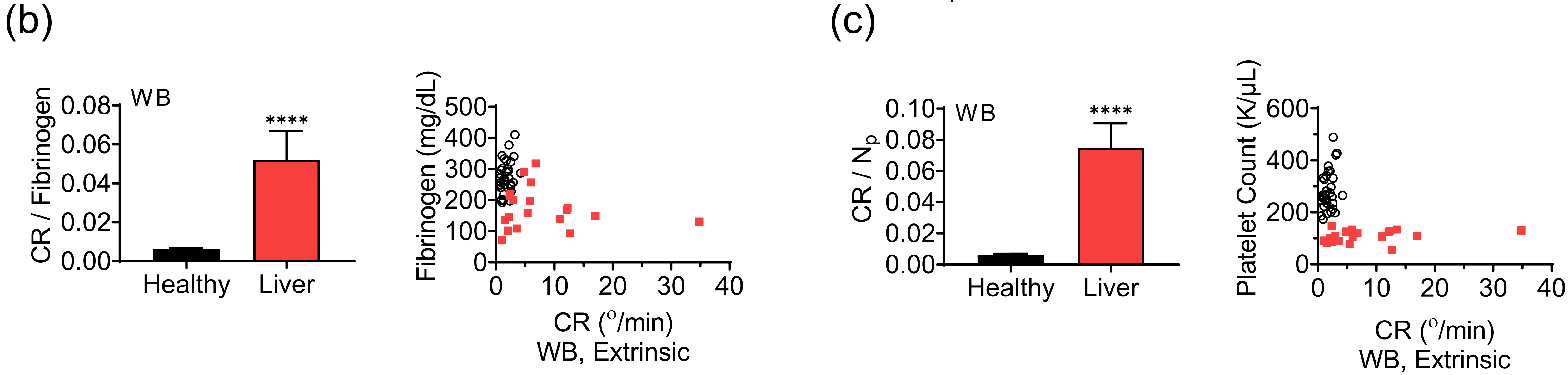

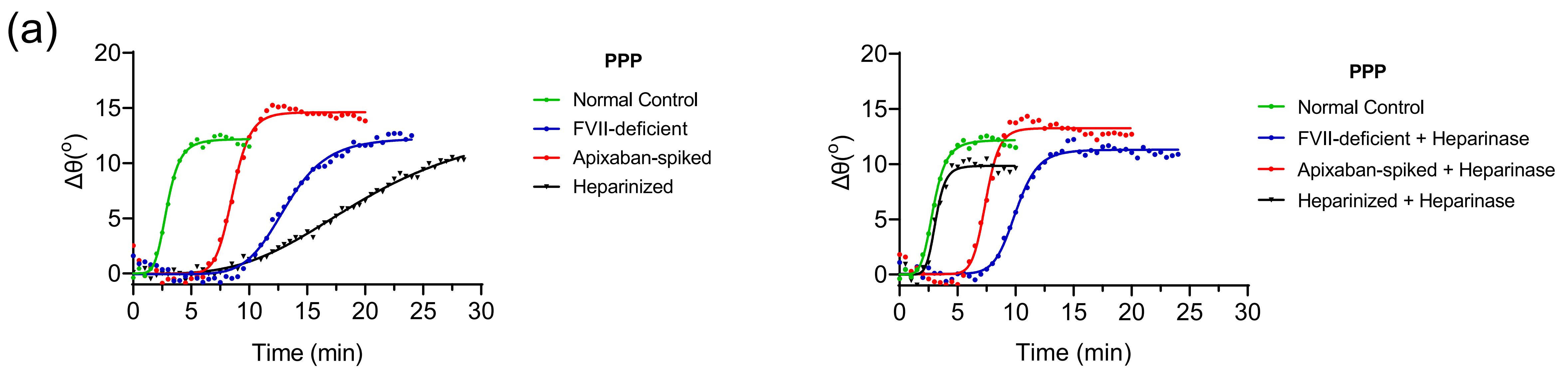

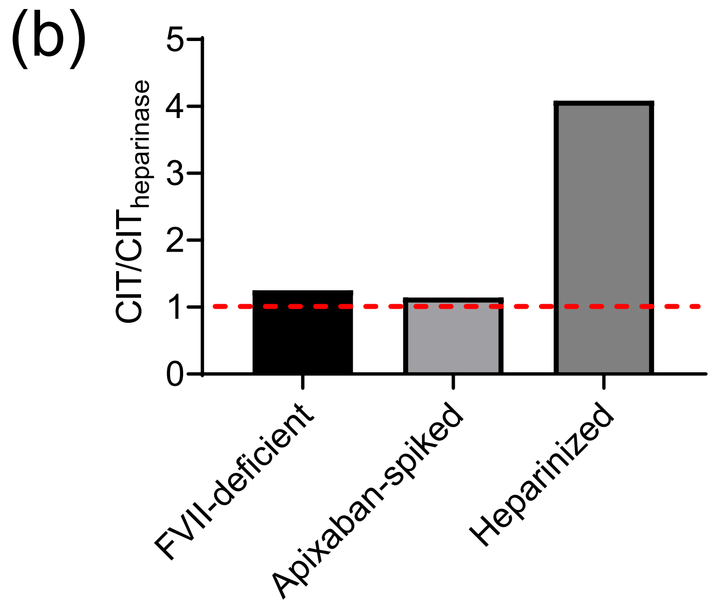

Supplementary Figure S9. (a) Mechanical tweezographs of plasma samples exhibiting prolonged clotting due to different causes, including factor deficiency (FVII-deficient) and anticoagulant treatment (apixaban-spiked and heparinized, left), and their corresponding heparinase-treated counterparts (right). (b) Ratio of CIT for each sample type before and after heparinase treatment, showing selective reversal of heparin-induced anticoagulation (n = 3-5).

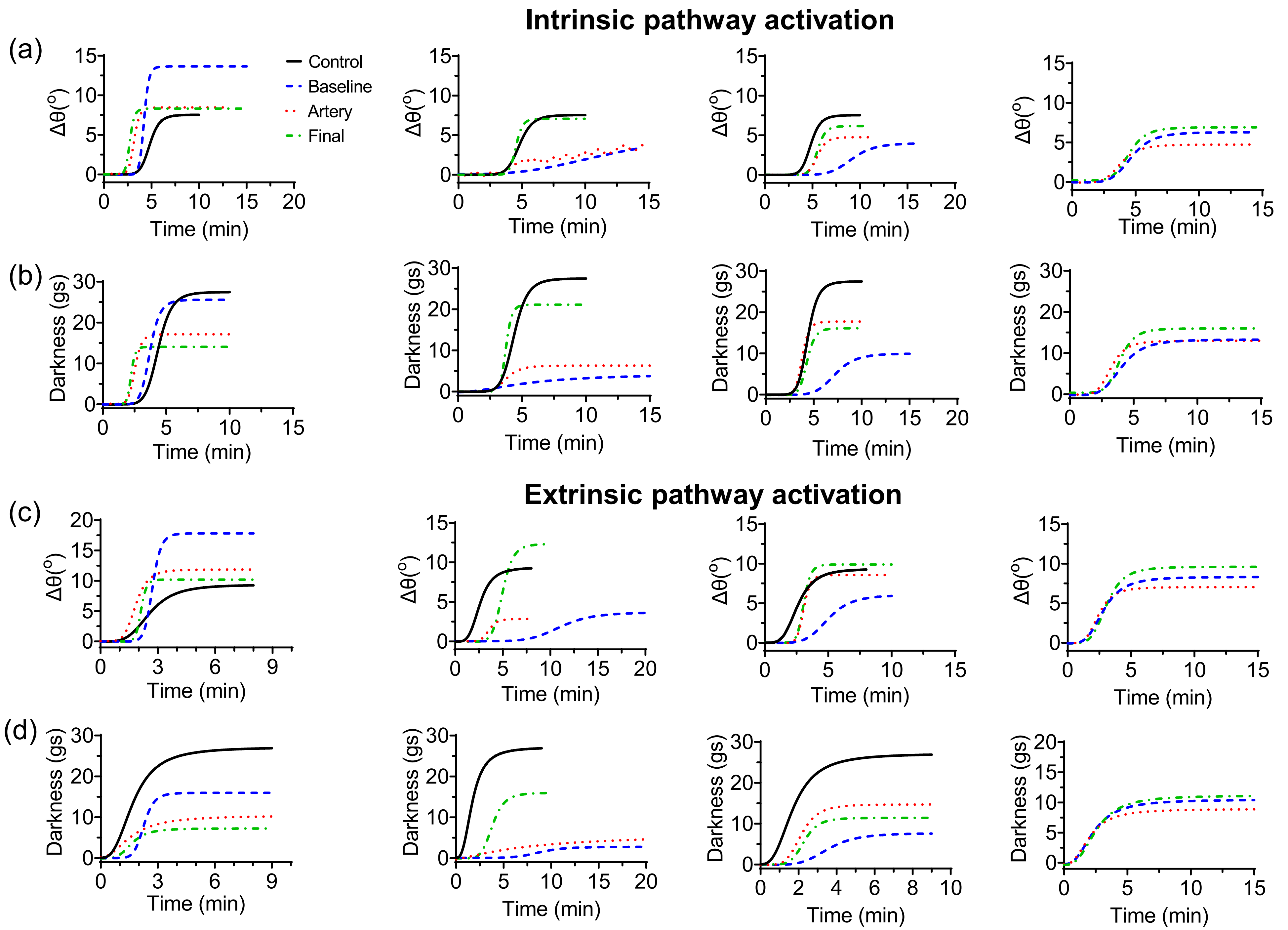

Supplementary Figure S10. Representative (first, second, and third columns) and averaged (fourth column, n = 6) mechanical (first and third rows) and photo-optical (second and fourth rows) tweezographs of liver transplant PPP samples activated via the intrinsic (a, b) or extrinsic (c, d) pathway.

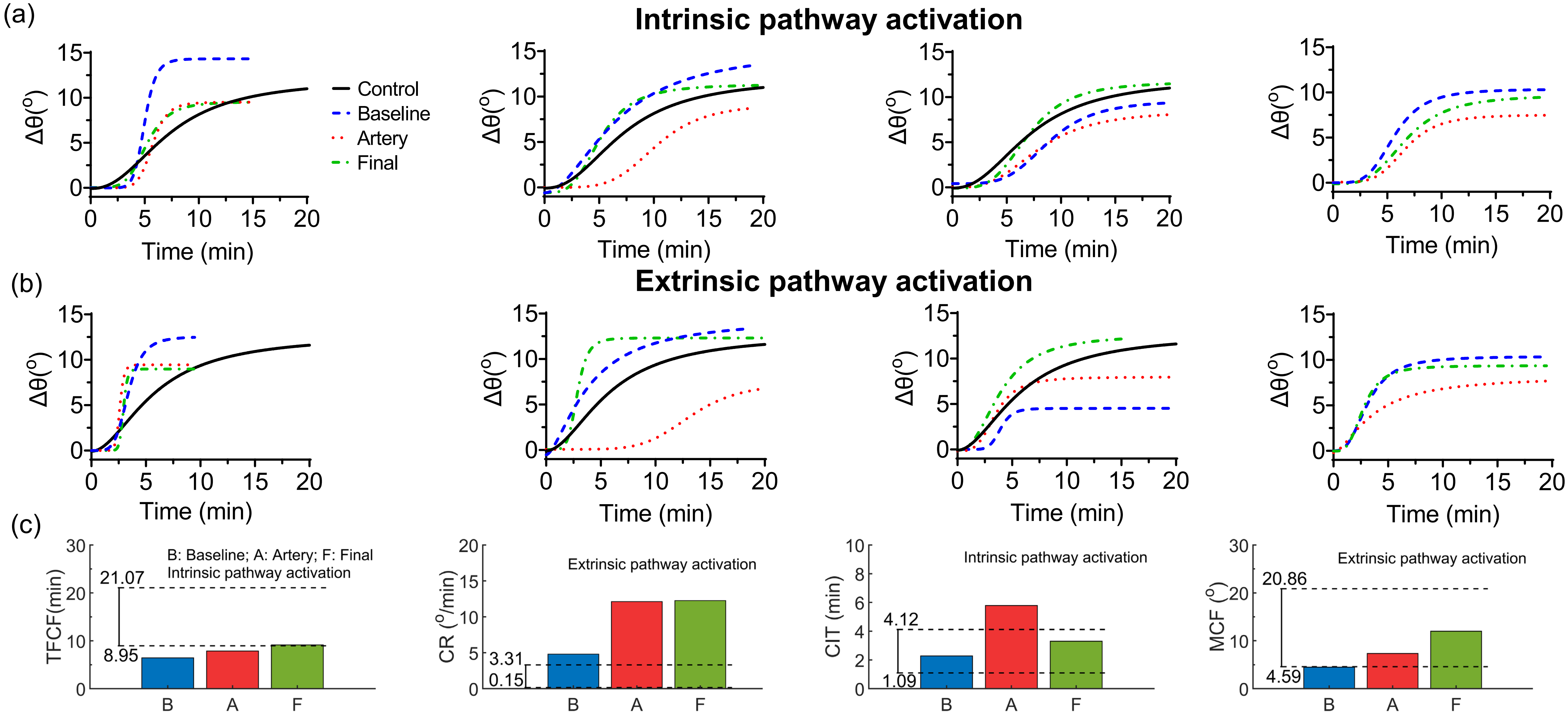

Supplementary Figure S11. Representative (first, second, and third columns) and averaged (fourth column, n = 6) mechanical tweezographs of liver transplant WB samples activated via the intrinsic (a) or extrinsic (b) pathway. (c) Parameters are shown for the following representative cases: hypercoagulability (first two plots); hypocoagulability (third plot); rebalanced hemostasis with reduced clot firmness at baseline time point (fourth plot).

Supplementary References

1. Association of Public Health Laboratories (APHL). Laboratory Test Verification and Validation Toolkit. 2024.
2. Emani S, Emani VS, Diallo FB, Dutta P, Matte GS, Nathan M, Ibla JC, Emani SM. Comparison of Thromboelastography Devices TEG(®)6S Point of Care Device vs. TEG(®)5000 in Pediatric Patients Undergoing Cardiac Surgery. *J Extra Corpor Technol*. 2022; **54**: 42-9. 10.1182/ject-42-49.
3. Sunnersjö L, Lindström H, Schött U, Törnquist N, Kander T. The precision of ROTEM EXTEM is decreased in hypocoagulable blood: a prospective observational study. *Thromb J*. 2023; **21**: 23. 10.1186/s12959-023-00468-5.
4. Anderson L, Quasim I, Steven M, Moise SF, Shelley B, Schraag S, Sinclair A. Interoperator and intraoperator variability of whole blood coagulation assays: a comparison of thromboelastography and rotational thromboelastometry. *J Cardiothorac Vasc Anesth*. 2014; **28**: 1550-7. 10.1053/j.jvca.2014.05.023.
5. Mpaili E, Tsilimigras DI, Moris D, Sigala F, Frank SM, Hartmann J, Pawlik TM. Utility of viscoelastic coagulation testing in liver surgery: a systematic review. *HPB*. 2021; **23**: 331-43.
6. Maria A, Lal BB, Khanna R, Sood V, Mukund A, Bajpai M, Alam S. Rotational thromboelastometry-guided blood component use in cirrhotic children undergoing invasive procedures: randomized controlled trial. *Liver International*. 2022; **42**: 2492-500.
7. Kumar R, Ng LX, Wong YJ, Tan CK, Wang LZ, Qiu TY, Wong B, Lin KW, Li JW, Kwek AB. Rotational thromboelastometry reduces the need for preemptive transfusion in cirrhosis: a randomized controlled trial (NCT: 05698134). *Journal of Clinical and Experimental Hepatology*. 2025; **15**: 102409.
